# Loss of function of *VCP/TER94* causes neurodegeneration

**DOI:** 10.1242/dmm.050359

**Published:** 2024-12-23

**Authors:** Kohei Tsumaki, Christian J. F. Bertens, Minoru Nakayama, Saya Kato, Yuki Jonao, Ayu Kuribayashi, Konosuke Sato, Shota Ishiyama, Momoko Asakawa, Riko Aihara, Yuki Yoshioka, Hidenori Homma, Hikari Tanaka, Kyota Fujita, Hitoshi Okazawa, Masaki Sone

**Affiliations:** ^1^ Department of Biomolecular Science, Faculty of Science, Toho University, Chiba 274-8510, Japan; ^2^ University Eye Clinic Maastricht, School for Mental Health and Neuroscience (MHeNs), Maastricht University, 6202AZ Maastricht, The Netherlands; ^3^ Department of Neuropathology, Institute of Science Tokyo, Tokyo 113-8510, Japan

**Keywords:** VCP, TER94, *Drosophila*, Frontotemporal dementia, Neurodegeneration, TDP43

## Abstract

Variants in several genes are linked to human frontotemporal lobar degeneration (FTLD) associated with TDP43- and/or ubiquitin-positive inclusions. However, it is not yet clear whether the underlying mechanism is a gain-of-function or a loss-of-function one. To answer this question, we used *Drosophila* expressing double-stranded RNA against the FTLD-associated gene *TER94* (an ortholog of *VCP*/p97) and found that the knockdown (KD) of this gene caused premature lethality, reduction in brain volume and alterations in the morphology of mushroom bodies. The changes caused by *TER94* KD were rescued by wild-type *TER94* but not by the human disease-linked A229E mutant, indicating that this mutant causes loss of function. Alterations were also observed in pupal brains and were partially rescued by co-expression of Mcm2, which is involved in control of the cell cycle, suggesting that dysregulation of neuronal proliferation caused the phenotypes. *TER94* KD also caused the disappearance of TBPH (an ortholog of TDP43/TARDBP) from nuclei. These data from *Drosophila* genetics suggest that VCP-linked FTLD is caused by loss-of-function of *VCP*.

## Teaser

**Summary:** Mutations in the *VCP* gene cause frontotemporal lobar degeneration. Our data from a *Drosophila* model including genetic rescue experiments revealed that loss-of-function of VCP/TER94 causes neurodegeneration.

## INTRODUCTION

Variants in several genes have been found to cause frontotemporal lobar degeneration (FTLD). Although genetic variants in the tau (also known as *MAPT*) gene cause pathological changes that are associated with the formation of tau-positive inclusions, variants in other genes, such as progranulin, VCP, CHMP2B and TDP43 (also known as TARDBP)*,* cause diseases associated with ubiquitin-positive and tau-negative inclusion bodies that also include TDP43 (Josephs, 2008; Olney et al., 2017). In particular, variants in VCP cause a specific type of FTLD, inclusion body myopathy associated with Paget disease of bone and frontotemporal dementia (IBMPFD) and amyotrophic lateral sclerosis (ALS), also referred to as multisystem proteinopathy type 1 (MSP1) (Watts et al., 2004; Pfeffer et al., 2022; Scarian et al., 2022).

VCP (also called TERA/p97/CDC48) is a member of the AAA ATPase family and is involved in diverse cellular functions. VCP contains two ATPase catalytic domains called the D1 and D2 domains and regulatory N-terminal and C-terminal domains. It forms a symmetric hexametric structure and couples ATP hydrolysis to multiple unrelated cellular activities through interactions with binding partners such as p47 (also known as PLEK) and the UFD1–NPL4 (also known as NPLOC4) complex (Woodman, 2003; Xia et al., 2016). For instance, VCP facilitates the translocation of misfolded proteins across the endoplasmic reticulum (ER) membrane in ER-associated degradation (ERAD) and mitochondria-associated degradation (Ye et al., 2001; Heo et al., 2010). VCP is implicated in membrane fusion events, including the assembly of the nuclear membrane, Golgi apparatus and transitional ER (Kondo et al., 1997; Roy et al., 2000; Hetzer et al., 2001). VCP is also involved in autophagy (Ju et al., 2009; Tresse et al., 2010; Ferrari et al., 2022).

Variants in the N-terminal domain, the D1 ATPase domain, the D2 ATPase domain and the N-D1 linker of VCP have been found to be associated with IBMPFD and ALS (Pfeffer et al., 2022; Scarian et al., 2022). However, VCP variants in the N-terminal domain or D1 ATPase domain have been suggested to elevate ATPase activity *in vitro* (Weihl et al., 2006; Halawani et al., 2009; Manno et al., 2010). Consistently, the expression of mutant VCP in cultured cells causes the accumulation of ubiquitin, decreases proteasome activity, impairs ERAD, elevates ER stress and eventually leads to cell death (Weihl et al., 2006; Ju et al., 2008; Gitcho et al., 2009). However, RNA interference (RNAi)-mediated knockdown (KD) of VCP induces the accumulation of ubiquitinated proteins and cytoplasmic vacuolization in *Drosophila* S2 cells and human HeLa cells (Wojcik et al., 2004), and ER vacuolization and impaired protein trafficking in COS-7 cells (Mimnaugh et al., 2006). Expression of dominant-negative VCP causes the accumulation of ubiquitinated proteins, ERAD impairment and swelling of the ER (Dalal et al., 2004). Therefore, it is unclear whether these variants cause disease phenotypes through gain of toxic function or loss of normal function.

At the animal level, transgenic and knock-in mouse models have been generated. Both types of mouse model show degeneration of the brain associated with ubiquitin- and TDP43-positive protein inclusions (Weihl et al., 2007; Badadani et al., 2010; Custer et al., 2010). These changes might be explained by gain of abnormal function. However, the critical issue with these mouse experiments was that they lacked rescue experiments that could exclude the artificial effects of mutant protein overexpression. Consistent with this notion, mice with conditional knockout of VCP in neurons show phenotypes similar to disease phenotypes, such as brain atrophy and TDP43 pathology (Wani et al., 2021). Although the expression of the VCP mutant R115C failed to rescue these phenotypes in the conditional knockout mice, it is also necessary to compare these effects with the rescuing effect of wild-type VCP to assess whether mutant VCP retains its rescuing ability.

Recently, we analyzed VCP T262A knock-in mice (Fujita et al., 2013; Homma et al., 2021). Our data revealed that developmental microcephaly was caused by G1/S arrest in neural stem cells. Insufficient DNA damage repair in neural stem cells was caused by elevated phosphorylation of mini-chromosome maintenance helicase (MCM)3, which is essential for the G1/S cell cycle transition. *In utero* gene therapy inducing the expression of wild-type VCP or nonphosphorylated mutant MCM3 successfully rescued DNA damage, neuronal necrosis and TDP43 aggregation in adult knock-in mice (Fujita et al., 2013; Homma et al., 2021). These data suggest that early-stage developmental abnormalities in G1/S cell cycle regulation underlie the pathology of FTLD.

In this study, we aimed to determine whether the disease-associated mutation in VCP is a loss-of-function or a gain-of-function mutation by using a *Drosophila* model. *Drosophila* has an evolutionally conserved ortholog of VCP called TER94 (Pintér et al., 1998)*.* This *Drosophila* ortholog was knocked down with RNAi, and we found that partial deficiency of TER94 caused neurodegeneration in the brain and eye. In the rescue experiment, normal *TER94* rescued these phenotypes. In contrast, the disease-linked A229E mutant barely rescued the phenotypes. Our data strongly suggest that loss of the cellular functions of VCP is the pathological basis of the disease linked to the *VCP* gene. Our data from the *Drosophila* model also suggest that defects in neuronal proliferation during development underlie the pathology of FTLD.

## RESULTS

### KD of *TER94* causes premature lethality

**Fig. 1.**
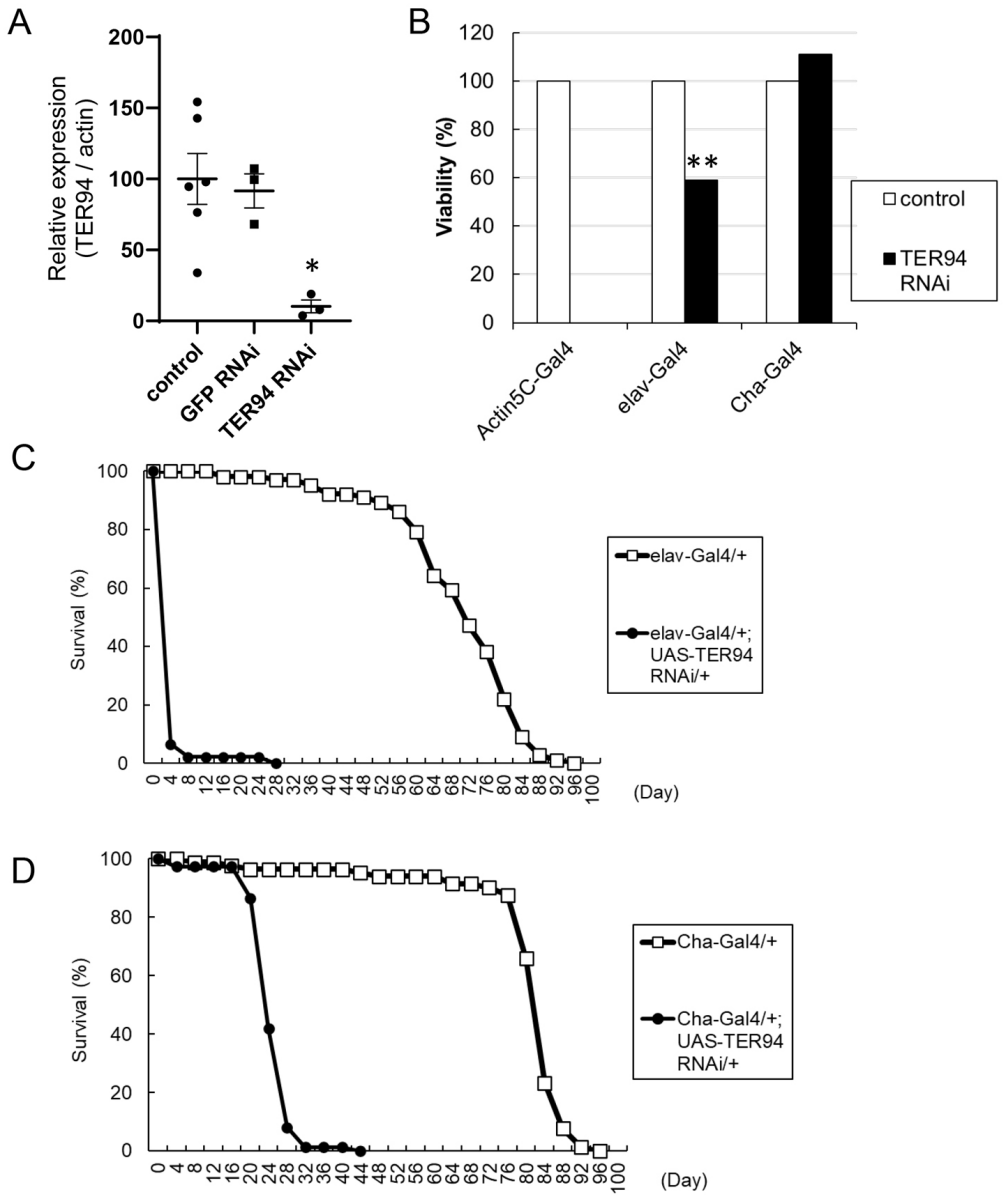
**Knockdown (KD) of *TER94* caused premature lethality.** (A) Expression of *TER94* mRNA normalized to that of *Actin5C* mRNA was examined in first-instar larvae by real-time PCR. *TER94* mRNA expression was significantly decreased by KD of *TER94* but not by KD of *GFP*. *P<0.05 (one-way ANOVA with Dunnett test). *n*=6 (control), *n*=3 [*GFP* RNA interference (RNAi)] and *n*=3 (*TER94* RNAi). (B) Viability of female flies during development from embryo to eclosion. Ubiquitous *Actin5C-Gal4*-driven KD of *TER94* resulted in lethality. Neuron-specific *elav-Gal4*-driven KD caused decreased viability. Cholinergic neuron-specific *Cha-Gal4*-driven KD did not affect viability. **P<0.01 (chi-square test). *n*=352 (*Actin5C*, control), *n*=377 (*Actin5C*, *TER94* RNAi), *n*=398 (*elav*, control), *n*=198 (*elav*, *TER94* RNAi), *n*=287 (*Cha*, control) and *n*=244 (*Cha*, *TER94* RNAi). (C) Lifespan of female adult flies after eclosion. Neuron-specific *elav-Gal4*-driven KD of *TER94* resulted in premature lethality. *n*=102 (control) and *n*=46 (*TER94* RNAi). (D) Cholinergic neuron-specific *Cha-Gal4*-driven KD of *TER94* also resulted in premature lethality, although the lifespan of these flies was longer than that of *elav-Gal4*-driven KD flies. *n*=86 (control) and *n*=74 (*TER94* RNAi).

We examined the phenotypes caused by RNAi-mediated KD of *TER94* driven by the combination of UAS-dsRNA and Gal4 drivers (Brand and Perrimon, 1993). First, we quantified the effect of KD by real-time quantitative PCR. Ubiquitous *Actin5C-Gal4*-driven KD of *TER94* resulted in a significant decrease in the transcription of *TER94* in whole first-instar larvae (Fig. 1A), whereas *Actin5C-Gal4*-driven KD of green fluorescent protein (*GFP*), which was used as a control, did not cause a decrease in *TER94* transcripts. Western blot analysis using an antibody against TER94 confirmed that *Actin5C-Gal4*-driven KD of *TER94* caused a decrease in the expression of the TER94 protein (Fig. S1A,B). The *Actin5C>TER94*-KD flies showed complete lethality during development (Fig. 1B). We also examined the effects of *elav-Gal4*- and *Cha-Gal4*-driven *TER94* KD*. elav-Gal4* induces weak expression in all neurons (Luo et al., 1994). *Cha-Gal4* contains a promoter of the *Choline acetyl transferase* (*Cha*; also known as *ChAT*) gene and induces expression in all cholinergic neurons, which constitute the majority of the excitatory neurons in the *Drosophila* nervous system (Kitamoto, 2001). *elav*>*TER94*-KD flies showed reduced viability during development (Fig. 1B), and almost all the flies died within 4 days after eclosion (Fig. 1C). KD of *GFP* driven by *elav-Gal4*, which was performed as a control experiment, did not affect viability or lifespan (Fig. S2A,B), suggesting that the expression of double-stranded RNA (dsRNA) itself does not have significant effects. *Cha-Gal4*-mediated *TER94* KD did not affect viability during development. However, most flies died 20-30 days after eclosion (Fig. 1D). Thus, *TER94* KD clearly affected the survival of the animals.

### *TER94* KD causes neurodegeneration in the brain and the compound eye

**Fig. 2.**
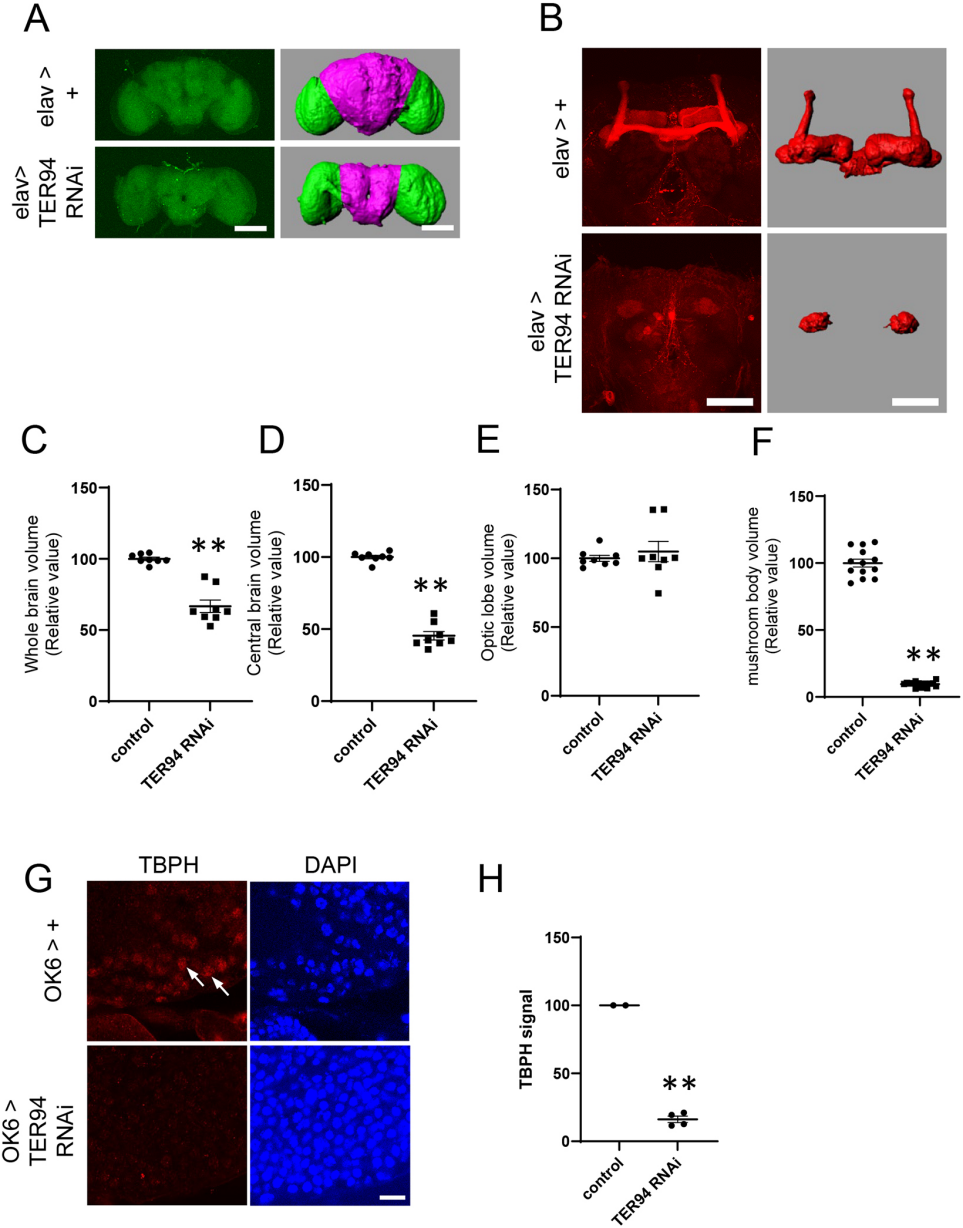
**Neuronal KD of *TER94* caused a reduction in brain volume, alterations in the morphology of mushroom bodies and disappearance of TBPH from nuclei.** (A) KD of *TER94* resulted in reduction in the volume of the central brain. Autofluorescence in projections of confocal images of the brains is shown on the left. Images reconstructed in Imaris software are shown on the right. The central brain is magenta, and the optic lobes are green. The control images shown in Fig. 2A and Fig. S5A are the same, as data were collected simultaneously. Scale bars: 100 μm. (B) KD of *TER94* resulted in alterations to the morphology of mushroom bodies. Projections of confocal images of mushroom bodies labeled by an anti-FasII antibody are shown on the left. Projection images reconstructed in Imaris are shown on the right. KD of *TER94* resulted in loss of lobe structures. The control images shown in Fig. 2B, Fig. 4A and Fig. S9A are the same, as data were collected simultaneously. Scale bars: 50 μm. (C) The volume of the whole brain was significantly reduced in KD flies. *n*=13 (control) and *n*=14 (*TER94* RNAi). (D) The volume of the central brain was significantly reduced in KD flies. *n*=13 (control) and *n*=14 (*TER94* RNAi). (E) The volume of the optic lobes was not changed. *n*=13 (control) and *n*=14 (*TER94* RNAi). (F) The volume of the mushroom body was significantly reduced in KD flies. *n*=8 (control) and *n*=8 (*TER94* RNAi). For C-F, **P<0.01 (unpaired two-tailed Student's *t*-test). (G) KD of *TER94* by *OK6-Gal4* in larval motor neurons. In the control neurons, TBPH protein was localized in the nucleus (arrows). When *TER94* was knocked down, the nuclear localization of TBPH decreased. DAPI, 4′,6-diamidino-2-phenylindole. Scale bar: 10 μm. (H) KD of *TER94* in motor neurons resulted in the disappearance of TBPH localization from the nucleus. **P<0.01 (unpaired two-tailed Student's *t*-test). *n*=2 (control) and *n*=4 (*TER94* RNAi).

Because *elav*>*TER94*-KD flies, in which *TER94* is knocked down in all neurons, died several days after eclosion, we examined the morphology of the brains of these flies immediately after eclosion. They were found to have a decreased brain volume compared with that of wild-type flies (Fig. 2A,C). Although the overall volume of the brain was decreased, its structure seemed not to be seriously disrupted. The volume of the central brain was decreased, but the optic lobes were not affected (Fig. 2D,E). The morphology of mushroom bodies was further examined by labeling mushroom body neurons by immunostaining using an anti-Fasciclin II (FasII; also known as Fas2) antibody (Crittenden et al., 1998; Awasaki et al., 2000). The morphology of mushroom bodies in the *TER94* KD flies was severely affected, resulting in loss of almost all their structures, including all the lobes (Fig. 2B,F). These data indicated that *TER94* KD caused neurodegeneration.

We also performed similar experiments on the compound eye. Compound eye-specific *GMR-Gal4*-driven KD of *TER94* resulted in progressive loss of pigments in the compound eye (Fig. S3A). Examination of the internal morphology of the compound eye revealed progressive deterioration of tissues accompanied by vacuolization and loss of rhabdomeres (Fig. S3B,D). The thickness of the eye was decreased at eclosion and in aged flies (Fig. S3C). These data indicated that *TER94* KD caused progressive tissue degeneration in the compound eye.

### *TER94* KD causes the disappearance of TBPH from nuclei

Next, we examined whether *TER94* KD affects the nuclear localization of TBPH, the *Drosophila* ortholog of mammalian TDP43 (Lukacsovich et al., 1999), because changes in the localization of TDP43 are a well-defined pathological feature of IBMPFD. We knocked down *TER94* in third-instar larval motor neurons in the ventral nerve cord using the *OK6-Gal4* driver (Shiraishi et al., 2014). Immunostaining with an antibody against TBPH revealed that the localization of TBPH in the nucleus was significantly diminished by *TER94* KD (Fig. 2G,H), but the amount of *TBPH* mRNA was not significantly changed (Fig. S4A). Western blotting also revealed that the level of TBPH protein was decreased by *TER94* KD (Fig. S4B).

### IBMPFD-associated mutant *TER94* A229E fails to rescue the KD phenotypes

Our data showed that decreased expression of *TER94* resulted in deterioration of neural tissues. Our findings raised the possibility that IBMPFD-associated mutations in VCP cause loss of its function and result in a dominant disease due to haplo-insufficiency or through a dominant-negative effect. To test this hypothesis, we examined whether the phenotypes of *TER94* KD flies could be rescued by additional expression of wild-type and disease-associated mutant *TER94*.

**Fig. 3.**
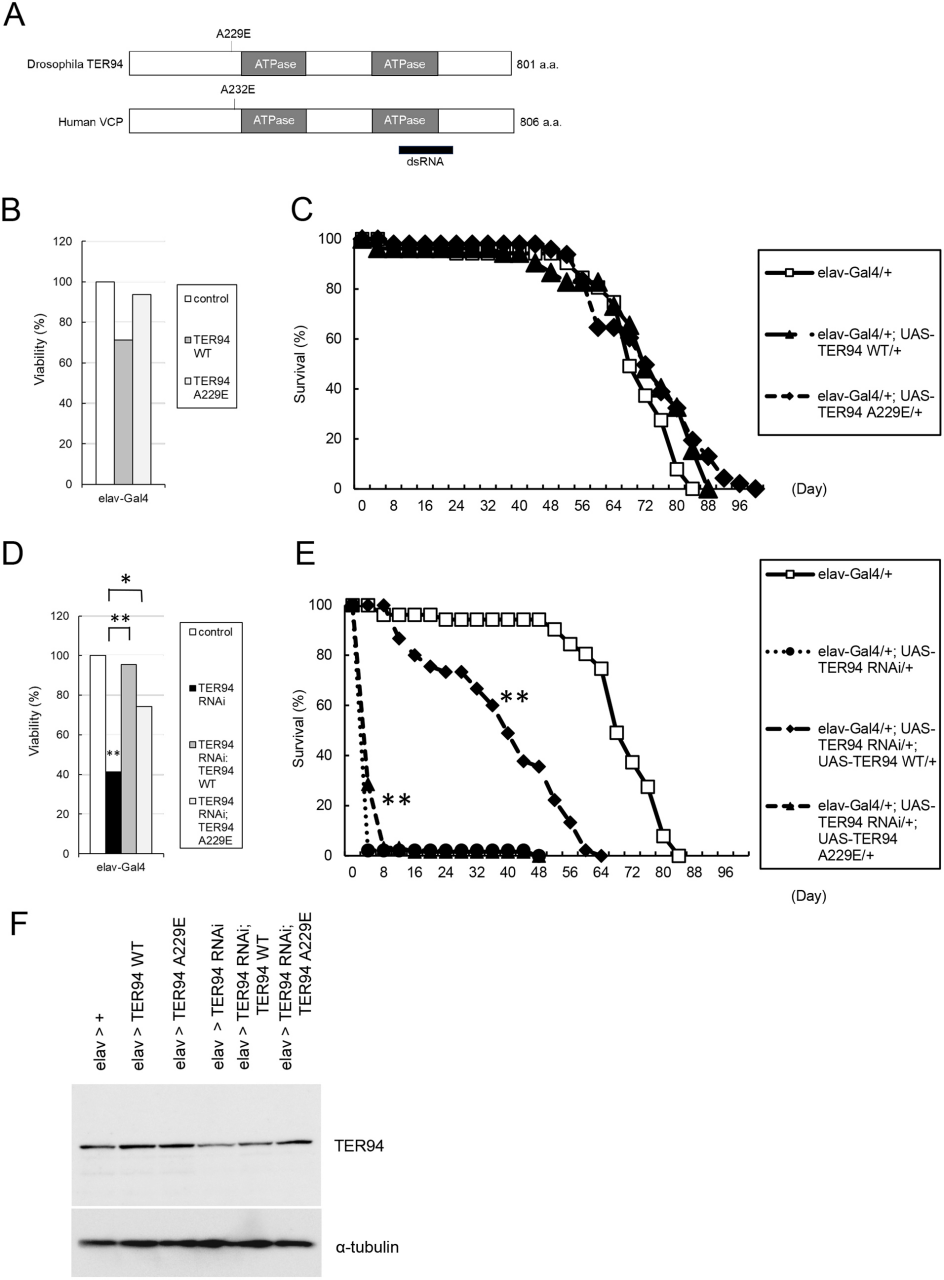
**Rescue of premature lethality in *TER94* KD flies by wild-type and mutant *TER94*.** (A) Schematic diagram showing the *Drosophila* TER94 (top) and human VCP (bottom) proteins. The position of the double-stranded RNA (dsRNA) used in the experiments is shown. a.a., amino acids. (B) Viability of flies during development from embryo to adult. Neuronal expression of wild-type and mutant *TER94* did not affect viability. *n*=287 (control), *n*=209 [*TER94* wild type (WT)] and *n*=146 (*TER94* A229E). (C) Lifespan after eclosion. Neuronal expression of wild-type and mutant *TER94* did not affect lifespan. *n*=52 (control), *n*=53 (*TER94* WT) and *n*=48 (*TER94* A229E). (D) Viability of flies during development from embryo to adult. Expression of both wild-type and mutant *TER94* rescued the reduction in viability caused by *TER94* KD. *n*=243 (*TER94* RNAi), *n*=295 (*TER94* RNAi; *TER94* WT) and *n*=81 (*TER94* RNAi; *TER94* A229E). *P<0.05, **P<0.01 (chi-square test). (E) Lifespan after eclosion. Although neuronal expression of wild-type *TER94* significantly rescued the premature lethality caused by *TER94* KD, expression of mutant *TER94* only slightly rescued the phenotypes. *n*=47 (*TER94* RNAi), *n*=45 (*TER94* RNAi; *TER94* WT) and *n*=63 (*TER94* RNAi; *TER94* A229E). **P<0.01 (log rank test compared with RNAi flies). (F) Western blot analysis of lysates of adult fly heads with an anti-TER94 antibody.

**Fig. 4.**
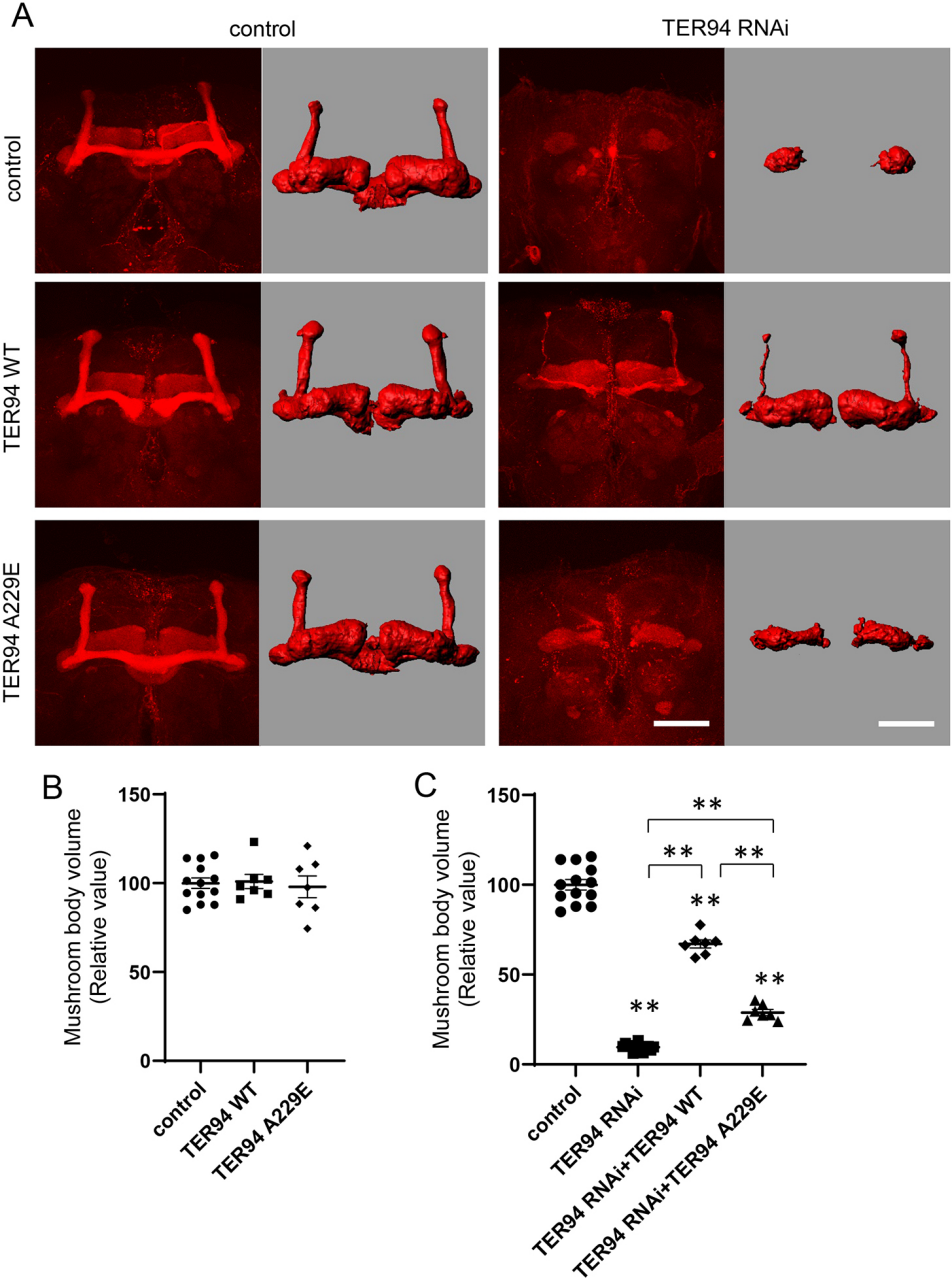
**Rescue of alterations in the morphology of mushroom bodies in *TER94* KD flies by wild-type and mutant *TER94*.** (A) Morphology of mushroom bodies. The left columns show projections of confocal images stained by an anti-FasII antibody. The right columns are images reconstructed in Imaris software. The control images shown in Fig. 2B, Fig. 4A and Fig. S9A are the same, as data were collected simultaneously. Scale bars: 50 μm. (B) Mushroom body volume was not affected by neuronal expression of wild-type or mutant *TER94*. *n*=13 (control), *n*=7 (*TER94* WT) and *n*=7 (*TER94* A229E). (C) The reduction in mushroom body volume caused by *TER94* KD was significantly rescued by neuronal expression of wild-type *TER94* but only slightly rescued by expression of mutant *TER94*. *n*=14 (*TER94* RNAi), *n*=7 (*TER94* RNAi; *TER94* WT) and *n*=7 (*TER94* RNAi; *TER94* A229E). **P<0.01 (one-way ANOVA with Games-Howell test).

We first examined the effect of ectopic expression of wild-type and A229E mutant *TER94* (Ritson et al., 2010) (Fig. 3A), because we could test their rescuing ability under the prerequisite that ectopic expression itself does not cause a phenotype. The site of the A229E mutation of *Drosophila TER94* is close to the D1 ATPase domain, and this mutation is a homolog of the A232E variant of human VCP. Ectopic expression of wild-type or mutant *TER94* by *elav-Gal4* did not affect viability (Fig. 3B), lifespan (Fig. 3C), central brain volume (Fig. S5A,B) or mushroom body volume (Fig. 4A,B). Similarities in lifespan among *elav-Gal4*>+, *elav-Gal4*>wild-type *TER94* and *elav-Gal4*>mutant *TER94* flies also confirmed the homogeneity of the genetic background in these flies (Fig. 3C). However, *GMR-Gal4*-driven expression of wild-type and mutant *TER94* caused eye degeneration and lethality, respectively (Fig. S6A). Quantitative PCR revealed that the expression of *GFP* driven by *GMR-Gal4* was far higher than that driven by *elav-Gal4* (Fig. S6B). This suggested that when *TER94* was expressed by *GMR-Gal4*, overexpression itself caused phenotypes. For this reason, we used only *elav-Gal4* in the rescue experiments.

We tested whether the phenotypes resulting from *TER94* KD driven by *elav-Gal4* could be rescued by additional expression of wild-type or mutant *TER94*. Both showed an ability to rescue the developmental semilethality of *TER94* KD (Fig. 3D). Whereas wild-type *TER94* showed a remarkable rescue effect on the decrease in lifespan, mutant A229E *TER94* showed a very weak rescue effect (Fig. 3E). Co-expression of *GFP* did not rescue the developmental semilethality or lifespan shortening, as was observed in the control experiment (Fig. S2C,D). Wild-type *TER94* had a remarkable rescue effect on the decreases in central brain volume and mushroom body volume, whereas mutant *TER94* showed a relatively weak rescue effect (Fig. 4A,C; Fig. S5A,C). Western blot analysis using an anti-TER94 antibody revealed that mutant TER94 expression was induced at a level comparable to that of wild-type TER94 expression (Fig. 3F; Fig. S7). These data indicated that the A229E mutation caused loss of the physiological functions of the *TER94* gene.

### KD phenotypes begin in the developmental stage

Our previous study investigating VCP knock-in mice revealed that a VCP mutation causes developmental microcephaly because of G1/S arrest in neural stem cells. We found that the phosphorylation of MCM3 and MCM2 was elevated in VCP T262A knock-in mice (Homma et al., 2021). MCM2 and MCM3 are components of a protein complex that forms the core of the replicative DNA helicase and is involved in the licensing of DNA replication origins (Remus et al., 2009; Li et al., 2015). Our previous study also suggested that early developmental abnormalities in G1/S cell cycle regulation underlie the pathology of FTLD because induced expression of wild-type VCP or nonphosphorylated mutant of MCM3 successfully rescued DNA damage, neuronal necrosis and TDP43 aggregation in adult knock-in mice (Homma et al., 2021).

Therefore, we examined whether overexpression of Mcm2 or Mcm3 rescues the *TER94* KD phenotypes. To test a prerequisite of the rescue experiment, we examined the effect of ectopic expression of Mcm2 and Mcm3 driven by neuronal *elav-Gal4*. Both did not have any effect on viability (Fig. S8A). However, when Mcm2 was overexpressed, ∼20% of the flies died within 4 days of eclosion. When Mcm3 was overexpressed, lifespan was shortened to ∼40 days (Fig. S8B). Therefore, we decided to test the rescuing ability of Mcm2 and Mcm3 before eclosion and in day 1 adult flies.

We examined whether developmental semilethality caused by *TER94* KD could be rescued. Expression of Mcm2 fully rescued fly viability, whereas expression of Mcm3 did not (Fig. S9B). We also examined whether the reduction in mushroom body volume could be rescued. Expression of Mcm2 significantly rescued the mushroom body phenotype, whereas expression of Mcm3 did not (Fig. S9A,C,D, Fig. S10A). Our real-time PCR data suggested that overexpression of *Mcm2* does not affect *TER94* KD itself (Fig. S10B). These data indicated that overexpression of Mcm2 partially rescued the phenotypes of *TER94* KD.

**Fig. 5.**
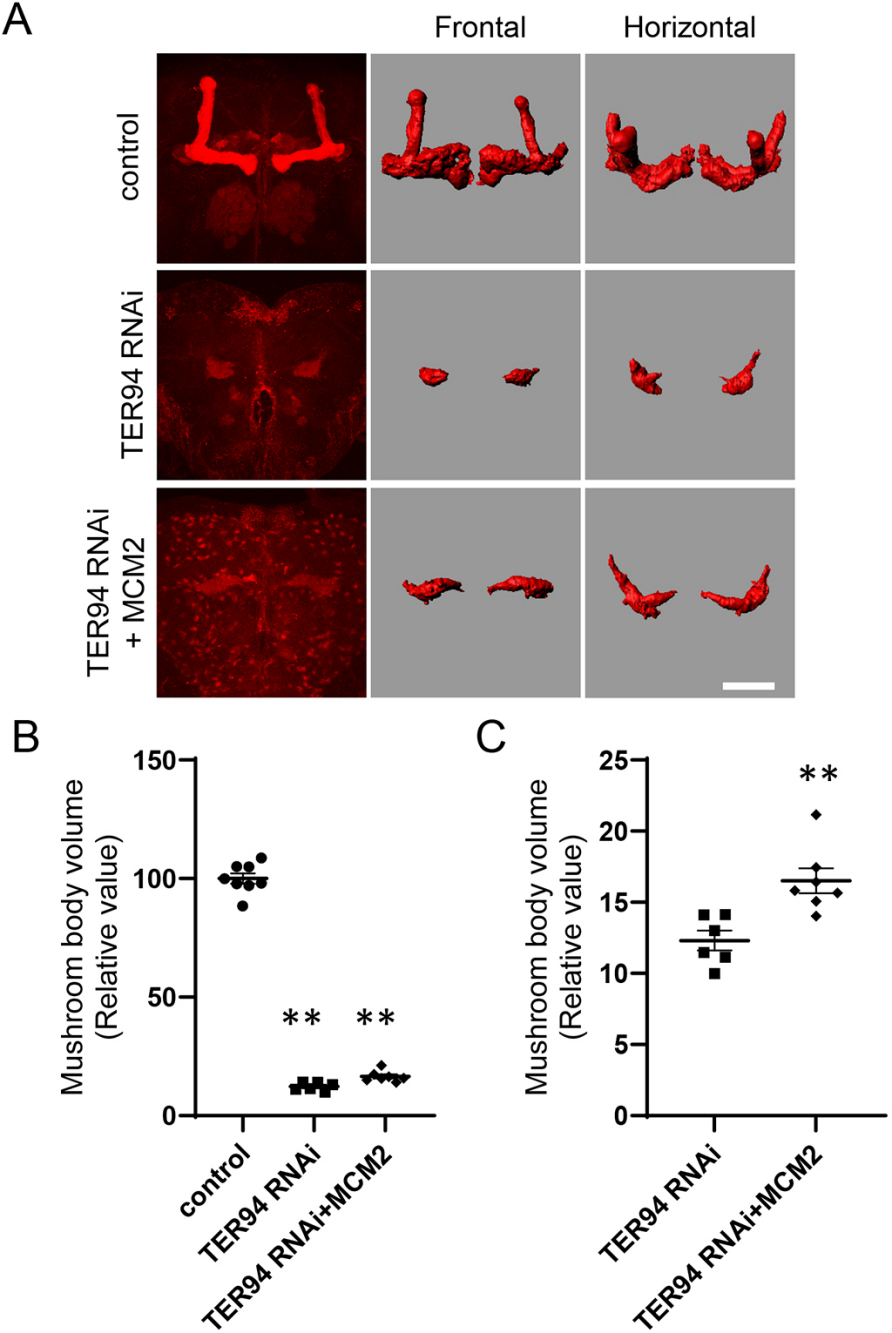
**Alterations in the morphology of mushroom bodies in pupae caused by *TER94* KD.** (A) Morphology of mushroom bodies of pupae 72 h after eclosion. The left column shows projections of confocal images stained by an anti-FasII antibody. The center and right columns are images reconstructed in Imaris software; the center column shows frontal images, and the right column shows horizontal images. Scale bar: 50 μm. (B) Pupal mushroom body volume was significantly reduced by *TER94* KD. *n*=8 (control), *n*=6 (*TER94* RNAi) and *n*=7 (*TER94* RNAi; Mcm2). **P<0.01 (one-way ANOVA with Games-Howell test). (C) The reduction in mushroom body volume was rescued by overexpression of Mcm2. **P<0.01 (one-way ANOVA with Games-Howell test).

Next, we examined whether the decrease in mushroom body volume resulted from abnormalities in the pupal developmental stage. We examined the mushroom body volume of pupae 72 h after puparium formation (APF). *TER94* KD caused marked reduction in mushroom body volume, which was significantly rescued by overexpression of Mcm2 (Fig. 5A-C). Thus, our data suggested that the phenotypes of *TER94* KD resulted from abnormalities in G1/S cell cycle transition in the developmental stage.

## DISCUSSION

Our data show that RNAi-mediated depletion of *TER94*, an ortholog of human *VCP*, caused brain degeneration, eye degeneration and premature lethality. KD driven by the ubiquitous and strong driver *Actin5C-Gal4* resulted in developmental lethality. *elav-Gal4*-mediated KD in all neurons resulted in developmental semilethality, decreased brain volume, decreased mushroom body volume and early death within 4 days of eclosion. Compound eye-specific KD mediated by *GMR-Gal4* caused severe degeneration of tissue. Remarkable cell loss and vacuolization were observed in adult flies just after eclosion. These pathological changes progressed rapidly toward complete deterioration of tissue structure. In addition, *TER94* KD caused a decrease in endogenous TBPH, an ortholog of TDP43, in the nuclei of larval motor neurons, and this could have been caused by loss of the physiological function of *TER94* in the nuclear entry of TBPH as previously suggested (Kushimura et al., 2018). These observations indicated that the KD of *TER94* recapitulates the pathological changes caused by VCP mutations in humans.

Importantly, the phenotypes of *TER94* KD flies were rescued by wild-type *TER94* but were rescued to a lesser extent by mutant *TER94*. All of the phenotypes caused by *elav-Gal4*-driven *TER94* KD were successfully rescued by co-expression of wild-type TER94, suggesting that these phenotypes were caused by KD of the *TER94* gene. Because our data showed that RNAi-mediated depletion of *TER94* caused neurodegeneration, we assessed the relevance of these phenotypes to the pathological changes caused by genetic mutations associated with IBMPFD. We performed genetic rescue experiments by inducing additional expression of wild-type and mutant *TER94*. Additional expression of wild-type *TER94* had remarkable rescue effects on semilethality, early death after eclosion, decreased central brain volume and decreased mushroom body volume. Among these measures, viability and central brain volume were restored to wild-type level, whereas lifespan and mushroom body volume were not completely restored to wild-type level. However, this could be attributed to the weak expression induced by *elav*-*Gal4*- and RNAi-mediated KD of the induced transcripts. Although our western blot analysis revealed that mutant TER94 expression was induced at a level comparable to that of wild-type TER94, it may be that the expression of both exogenous and endogenous TER94 varies among animals. This made it difficult to assess how much of the mRNA or protein was overexpressed. The fact that our phenotypic data clearly revealed that exogenous co-expression of wild-type TER94 with the dsRNA resulted in significant recovery of the KD-induced phenotypes suggests that these rescuing effects have been caused by additive expression of exogenous TER94, which may have caused the *TER94* expression level to surpass the threshold dosage for generating the mutant phenotypes. In addition, our experiments compared the effects of co-expression of wild-type *TER94*, which resulted in significant rescue effects, with the those of co-expression of mutant *TER94* or *GFP*, which resulted in weak or no rescue effects. This comparison ruled out the possibility that titration of UAS caused the KD to be less effective, resulting in rescuing effects.

In our rescue experiments, the A229E mutant *TER94* rescued only weakly the phenotypes of *TER94* KD. If A229E mutant *TER94* newly gains a toxic function, the phenotypes would have been worsened in the rescue experiments. However, we did not observe such results in our experiments. Thus, our rescue experiments indicated that FTLD caused by the A229E mutation is due to loss of function of the TER94 protein. These findings further suggested that human IBMPFD is caused by loss of function of VCP due to the A229E mutation.

Our results are consistent with the phenotypes of conditional VCP knockout mice. Neuronal VCP loss of function results in phenotypes including cortical brain atrophy and the formation of TDP43 inclusions, resembling the pathology of VCP-associated disease (Wani et al., 2021). The finding that induced expression of R115C mutant VCP in conditional knockout mice fails to rescue the knockout phenotypes is consistent with the results of our rescue experiments, although it is necessary to compare the effect of this mutant with that of wild-type VCP to assess its rescue ability.

All VCP mutations associated with human IBMPFD are missense mutations, and they are located in the N-terminal domain or the D1 ATPase domain in many cases (Pfeffer et al., 2022; Scarian et al., 2022). Previous studies suggested that disease mutations alter the conformations of the N-terminal domain and the D1 ATPase domain (Tang et al., 2010; Tang and Xia, 2013). The N-terminal domain is suggested to bind with co-factors and to link ATP hydrolysis with the unfolding or disassembly of substrate proteins (Uchiyama et al., 2002; Dreveny et al., 2004). Thus, disease mutations may alter co-factor binding and co-factor-regulated ATP hydrolysis (Zhang et al., 2015). These findings could provide links between missense mutations and loss of physiological functions.

Our data suggest that the phenotypes of *TER94* KD began in the pupal developmental stage because *elav*>*TER94*-KD pupae showed a decreased mushroom body volume. This developmental defect was suggested to be at least partly caused by G1/S arrest in neuroblasts because the pupal phenotype was rescued by overexpression of Mcm2. Although *elav*>*TER94*-KD flies showed markedly decreased mushroom body volume, the mushroom bodies seemed to be almost specifically affected, because the overall morphology of the brain was not severely disrupted and the optic lobe volume was not affected. This almost specific loss of mushroom bodies is similar to that reported in a previous study in which the proliferation of neuroblasts was blocked by hydroxyurea, which interferes with DNA synthesis (Sweeney et al., 2012). In our previous study of VCP knock-in mice, the phosphorylation of MCM2 and MCM3 was found to be elevated, and these molecules were found to be involved in G1/S arrest in neural stem cells (Homma et al., 2021). Our previous work suggested that phosphorylation of MCM2 was elevated in VCP knock-in mice. If mammalian VCP and *Drosophila* TER94 share similar molecular functions, *TER94* KD may cause hyperphosphorylation of Mcm2, and this could be rescued by overexpression of Mcm2. Despite our knock-in mouse study identifying MCM3, but not MCM2, as a critical factor in G1/S arrest, our rescue experiments identified Mcm2, but not Mcm3, as a causative factor of the phenotypes of *TER94* KD. Although these data suggest the evolutionally conserved importance of the hexametric MCM complex, which consists of MCM2-7 and plays critical roles in DNA replication origin licensing, in the causative mechanisms of VCP-associated disease that affect the proliferation of neural stem cells, the role of each subunit may be different between *Drosophila* and mammals. In our previous study of knock-in mice, gene therapy in the developmental stage rescued adult phenotypes (Homma et al., 2021). Thus, our *Drosophila* data also support the conclusion that pathological changes in the cell cycle in neural stem cells in early development underlie late-onset FTLD pathology.

In conclusion, our study suggests that loss of function rather than functional gain of function of *TER94/VCP* is linked to neurodegeneration. Our genetic rescue experiments further revealed that loss of VCP function underlies the pathogenetic mechanisms of the dominantly inherited disease IBMPFD.

## MATERIALS AND METHODS

### Fly genetics

Flies were raised on yeast–cornmeal medium (7.5% cornmeal, 3.8% yeast, 9.4% glucose, 3.0% wheat germ, 0.24% n-butyl p-hydroxybenzoate, 0.09% calcium chloride, 1.1% potassium tartrate hemihydrate and 0.9% agar). All fly stocks were maintained at 25°C under a 12:12 h light–dark cycle. All recombinant DNA experiments were approved by the Toho University Safety Committee for Recombinant DNA Experiments (20-454). Canton-S was used as the wild type. *UAS-TER94*-WT and *UAS-TER94*-A229E fly strains were a gift from Dr J. P. Taylor (St Jude Children's Research Hospital, Memphis, TN, USA). The *UAS-TER94*-KD strain (P{GD9777}v24354) was obtained from the Vienna *Drosophila* Resource Center. The *UAS-GFP*-KD strain (GFP-IR-1) was obtained from the National Institute of Genetics (NIG)-FLY stock center. The *UAS-Mcm2* strain (F001810) was obtained from the FlyORF Zurich ORFeome Project (Bischof et al., 2013). Expression of Mcm3 was induced by the UAS element in the P(EPg)HP10020 transgene in a fly strain that was obtained from Bloomington *Drosophila* Stock Center (stock number 21855).

### Quantitative PCR

For measurement of *TER94* expression by quantitative PCR, ten whole first-instar larvae, ten compound eyes from day 1 female adults, ten brains and ventral nerve cords from wandering third-instar larvae, and five to ten brains from day 1 female adults were collected and pooled in 50 μl RNA later solution (Merck) to obtain one sample. The samples were incubated at 4°C overnight and then stored at −30°C. The larvae were homogenized using Biomasher II (Nippi). Total RNA was isolated with an RNeasy mini kit (Qiagen), and cDNA was synthesized with a ReverTra Ace qPCR RT kit (Toyobo). Quantitative PCR analysis was performed with a 7500 Real-time PCR instrument (Applied Biosystems) using a *TER94* Taqman probe and primers for exon boundary 2 to 3 (Dm01844813_g1), an *Mcm3* Taqman probe and primers for exon boundary 1 to 2 (Dm01813006_g1), a *TBPH* Taqman probe and primers for exon boundary 4 to 5 (Dm01820179_g1), an *Actin5C* Taqman probe and primers (Sone et al., 2009), and Taqman Universal Master Mix II, no UNG (Thermo Fisher Scientific). To measure the expression of *GFP*, RNA was isolated from whole *Actin5C-Gal4*>*GFP* first-instar larvae, the brains of *elavGal4*>*GFP*, *Cha-Gal4*>*GFP* and +>*GFP* adults, the ventral nerve cords of *OK6-GFP*>*GFP* wandering third-instar larvae, and the compound eyes of *GMR-Gal4*>*GFP* adults. Quantitative PCR was performed on a 7300 Real-time PCR instrument (Applied Biosystems) using *GFP* primers (forward primer, 5′-TCTGTCTCCGGTCAAGGTGAAG-3′; reverse primer, 5′-GGCATGGCAGACTTGAAAAAG-3′) and a *Ras64B* Taqman Probe and primers for exon boundary 2 to 3 (Dm01822072_g1). *Ras64B* was used instead of *Actin5C* because expression of Actin5C was different between heads and compound eyes.

### Measurement of viability and lifespan

Viability and lifespan were measured as previously described (Furotani et al., 2018). All chromosomes were introduced into the same genetic background by consecutive backcrossing for more than three generations. The viability of female flies was measured by counting the number of eclosed flies on the day of eclosion and comparing it with the number of flies that had balancer chromosomes. For the measurement of lifespan, one to 20 female flies were reared at 25°C in a standard plastic vial.

### Measurement of brain volume and mushroom body volume

To measure the volume of the brains and mushroom bodies, dissection and immunostaining of pupal and adult brains were performed essentially as previously described (Saito et al., 2021). Female pupae were collected at 72 h APF, and female adult flies were collected at 1 day after eclosion. The flies were rinsed in 70% ethanol for 30 s. Then, their brains were dissected in phosphate-buffered saline (PBS). The brains were fixed in 4% paraformaldehyde in PBS for 1 h on ice. The samples were incubated with mouse Mab 1D4 anti-Fasciclin II monoclonal antibody (Developmental Studies Hybridoma Bank, Iowa University, Iowa City, IA, USA; diluted 1:50) in PBS containing 0.4% Triton X-100 overnight at 4°C. The samples were then incubated with a Cy3-conjugated anti-mouse IgG antibody (Jackson ImmunoResearch; diluted 1:200), mounted with Vectashield with DAPI (Vector Laboratories, H-1200). A coverslip was mounted on the other coverslips as pillows. Images were collected with a confocal microscope (Olympus, FV-1000) equipped with a UPLSAPO 20× objective (0.75 NA) and a UPLSAPO 60× oil-immersion objective (1.35 NA). Cy3 signals and green autofluorescence signals were scanned separately. Signals from 2 μm consecutive optical sections were collected from the entire brain. Photographs were reconstructed, and the volumes of the brains and mushroom bodies were measured using Imaris software (Zeiss).

### Analysis of the compound eye

Images of the compound eye were collected with an MZ16 microscope (Leica) equipped with an IC80HD camera (Leica). The internal structures of the compound eyes were examined by preparing semithin sections as previously described (Sone et al., 2009; Arimoto et al., 2020). The heads of female flies were collected in the daytime, cut longitudinally into halves and fixed in fixative (2.5% glutaraldehyde and 2% formaldehyde buffered with 0.1 M sodium cacodylate to pH 7.3) for more than 2 h at room temperature. They were then rinsed with 4% sucrose solution in the same buffer for 5 min three times on ice, postfixed with 1% osmium tetroxide in the same buffer for 1.5 h on ice, and rinsed with distilled water for 5 min three times on ice. Then, the samples were dehydrated in 50% ethanol, 70% ethanol and 90% ethanol each for 5 min each on ice, in dry ethanol for 5 min three times, and in propylene oxide for 5 min twice. The samples were then incubated with a 1:1 mixture of propylene oxide and Epon (TAAB) overnight and embedded in Epon. Semithin sections (500 nm thick) were cut with a diamond knife using an ultramicrotome (Leica). The sections were stained with Toluidine Blue, and photographs were taken using an Axioskop2 microscope (Zeiss) equipped with a DP73 camera (Olympus). The number of rhabdomeres in each ommatidium was counted by examining these photos. For the measurement of the thickness of the retina, paraffin sections of the compound eye were prepared as previously described (Sone et al., 2009). The heads of female flies were collected, the probosces were removed, and the samples were fixed in 4% paraformaldehyde in PBS overnight. The samples were then dehydrated with 70% and 90% ethanol for 15 min; 35% n-butanol, 50% ethanol and 15% water for 15 min; 55% n-butanol, 40% ethanol and 5% water for 15 min; 75% n-butanol and 25% ethanol for 15 min; and then 100% n-butanol for 15 min. Subsequently, the samples were embedded in paraffin, and 6 μm frontal sections of the entire brain were prepared and stained with Hematoxylin and Eosin. Images were captured using an Axioskop2 microscope (Zeiss) equipped with a DP73 camera (Olympus), and the thickness of the retina was measured using CellSens Dimension software (Olympus).

### Immunostaining of larval ventral nerve cords

Dissection and immunostaining of larval ventral nerve cords were performed essentially as previously described (Furotani et al., 2018). Female wandering third-instar larvae were collected, dissected and fixed for 20 min in 4% paraformaldehyde in PBS. The fixed larvae were subsequently incubated at 4°C overnight with an antibody against TBPH (Lin et al., 2011; diluted 1:200) in PBS containing 0.2% Tween 20 and 5% skim milk. The samples were then labeled with a secondary antibody conjugated to Cy3 (Jackson ImmunoResearch). The ventral nerve cords were subsequently dissected and mounted with Vectashield with DAPI (Vector Labs, H-1200). The samples were observed using a confocal microscope (Nikon, AX) with a 60× oil-immersion objective lens (1.42 NA). Single-plane images were obtained. The signal intensities in the nuclei were measured using CellSens Dimension software (Olympus).

### Western blot analysis

Female adult flies at day 1 after eclosion were collected, and their heads were isolated using forceps. The anterior third of wandering third-instar larvae containing the brain and the ventral nerve cord were collected. Otherwise, first-instar larvae were collected. The samples were then frozen in liquid nitrogen. Thirty heads or larvae were collected in SDS-PAGE sample buffer (125 mM Tris-HCl, pH 6.8, 4% SDS, 2.5% 2-mercaptoethanol, 10% glycerol and 0.005% Bromophenol Blue) containing Complete Protease Inhibitor (Sigma-Aldrich) and pooled to obtain one sample. The heads were homogenized using Biomasher II (Nippi), boiled, and electrophoresed on 5-20% gradient SDS polyacrylamide gels. Protein lysates from approximately four heads were loaded in each lane. The gel was electroblotted onto an Immobilon P membrane (Millipore). The membrane was blocked in TBST (10 mM Tris-HCl, pH 8.0, 62.5 mM NaCl, 0.05% Tween 20) containing 5% skimmed milk for 1 h, and incubated with rabbit anti-VCP antibody (Cell Signaling Technology, 2648S; diluted 1:300), rabbit anti-TBPH antibody (a gift from Dr C.-K. James Shen, Academia Sinica, Taipei, Taiwan; diluted 1:1000) or mouse anti-α tubulin antibody (Sigma-Aldrich, T6199; diluted 1:4000). Then, the membrane was incubated with horseradish peroxidase (HRP)-conjugated anti-rabbit IgG antibody (GE Healthcare, NA9345V; diluted 1:2500) or anti-mouse IgG antibody (GE Healthcare, NA931VD; diluted 1:5000) for 1 h. The signal was detected with an ECL prime system (Amersham) and LAS-4000 (GE Healthcare).

### Statistics

All data are shown as the mean±s.e.m. We performed statistical analysis using SPSS 27 software. When the ratios of eclosed fliles were compared, we used a chi-square test. When the averages of two groups were compared, we used a unpaired two-tailed Student's *t*-test. When the averages of more than two groups were compared, we used one-way ANOVA with post hoc tests. As a post hoc test, we used the Games-Howell test if the variance was significantly different among the genotypes. Otherwise, we used the Dunnett test.

## Supplementary Material

10.1242/dmm.050359_sup1 Supplementary information

## References

[DMM050359C1] Arimoto, E., Kawashima, Y., Choi, T., Unagami, M., Akiyama, S., Tomizawa, M., Yano, H., Suzuki, E. and Sone, M. (2020). Analysis of a cellular structure observed in the compound eyes of *Drosophila white; yata* mutants and *white* mutants. *Biol. Open* 9, bio047043. 10.1242/bio.047043 31862863 PMC6994944

[DMM050359C2] Awasaki, T., Saito, M., Sone, M., Suzuki, E., Sakai, R., Ito, K. and Hama, C. (2000). The *Drosophila* trio plays an essential role in patterning of axons by regulating their directional extension. *Neuron* 26, 119-131. 10.1016/S0896-6273(00)81143-5 10798397

[DMM050359C3] Badadani, M., Nalbandian, A., Watts, G. D., Vesa, J., Kitazawa, M., Su, H., Tanaja, J., Dec, E., Wallace, D. C., Mukherjee, J. et al. (2010). VCP associated inclusion body myopathy and paget disease of bone knock-in mouse model exhibits tissue pathology typical of human disease. *PLoS ONE* 5, e13183. 10.1371/journal.pone.0013183 20957154 PMC2950155

[DMM050359C4] Bischof, J., Björklund, M., Furger, E., Schertel, C., Taipale, J. and Basler, K. (2013). A versatile platform for creating a comprehensive UAS-ORFeome library in *Drosophila*. *Development* 140, 2434-2442. 10.1242/dev.088757 23637332

[DMM050359C5] Brand, A. H. and Perrimon, N. (1993). Targeted gene expression as a means of altering cell fates and generating dominant phenotypes. *Development* 118, 401-415. 10.1242/dev.118.2.401 8223268

[DMM050359C6] Crittenden, J. R., Skoulakis, E. M. C., Han, K.-A., Kalderon, D. and Davis, R. L. (1998). Tripartite mushroom body architecture revealed by antigenic markers. *Learn. Mem.* 5, 38-51. 10.1101/lm.5.1.38 10454371 PMC311260

[DMM050359C7] Custer, S. K., Neumann, M., Lu, H., Wright, A. C. and Taylor, J. P. (2010). Transgenic mice expressing mutant forms VCP/p97 recapitulate the full spectrum of IBMPFD including degeneration in muscle, brain and bone. *Hum. Mol. Genet.* 19, 1741-1755. 10.1093/hmg/ddq050 20147319

[DMM050359C8] Dalal, S., Rosser, M. F. N., Cyr, D. M. and Hanson, P. I. (2004). Distinct roles for the AAA ATPases NSF and p97 in the secretory pathway. *Mol. Biol. Cell* 15, 637-648. 10.1091/mbc.e03-02-0097 14617820 PMC329284

[DMM050359C9] Dreveny, I., Kondo, H., Uchiyama, K., Shaw, A., Zhang, X. and Freemont, P. S. (2004). Structural basis of the interaction between the AAA ATPase p97/VCP and its adaptor protein p47. *EMBO J.* 23, 1030-1039. 10.1038/sj.emboj.7600139 14988733 PMC380986

[DMM050359C10] Ferrari, V., Cristofani, R., Tedesco, B., Crippa, V., Chierichetti, M., Casarotto, E., Cozzi, M., Mina, F., Piccolella, M., Galbiati, M. et al. (2022). Valosin Containing Protein (VCP): a multistep regulator of autophagy. *Int. J. Mol. Sci.* 23, 1939. 10.3390/ijms23041939 35216053 PMC8878954

[DMM050359C11] Fujita, K., Nakamura, Y., Oka, T., Ito, H., Tamura, T., Tagawa, K., Sasabe, T., Katsuta, A., Motoki, K., Shiwaku, H. et al. (2013). A functional deficiency of TERA/VCP/p97 contributes to impaired DNA repair in multiple polyglutamine diseases. *Nat. Commun.* 4, 1816. 10.1038/ncomms2828 23652004 PMC4543262

[DMM050359C12] Furotani, K., Kamimura, K., Yajima, T., Nakayama, M., Enomoto, R., Tamura, T., Okazawa, H. and Sone, M. (2018). Suppression of the synaptic localization of a subset of proteins including APP partially ameliorates phenotypes of the *Drosophila* Alzheimer's disease model. *PLoS ONE* 13, e0204048. 10.1371/journal.pone.0204048 30226901 PMC6143267

[DMM050359C13] Gitcho, M. A., Strider, J., Carter, D., Taylor-Reinwald, L., Forman, M. S., Goate, A. M. and Cairns, N. J. (2009). VCP mutations causing frontotemporal lobar degeneration disrupt localization of TDP-43 and induce cell death. *J. Biol. Chem.* 284, 12384-12398. 10.1074/jbc.M900992200 19237541 PMC2673306

[DMM050359C14] Halawani, D., Leblanc, A. C., Rouiller, I., Michnick, S. W., Servant, M. J. and Latterich, M. (2009). Hereditary inclusion body myopathy-linked p97/VCP mutations in the NH2 domain and the D1 ring modulate p97/VCP ATPase activity and D2 ring conformation. *Mol. Cell. Biol.* 29, 4484-4494. 10.1128/MCB.00252-09 19506019 PMC2725746

[DMM050359C15] Heo, J.-M., Livnat-Levanon, N., Taylor, E. B., Jones, K. T., Dephoure, N., Ring, J., Xie, J., Brodsky, J. L., Madeo, F., Gygi, S. P. et al. (2010). A stress-responsive system for mitochondrial protein degradation. *Mol. Cell* 40, 465-480. 10.1016/j.molcel.2010.10.021 21070972 PMC2998070

[DMM050359C16] Hetzer, M., Meyer, H. H., Walther, T. C., Bilbao-Cortes, D., Warren, G. and Mattaj, I. W. (2001). Distinct AAA-ATPase p97 complexes function in discrete steps of nuclear assembly. *Nat. Cell Biol.* 3, 1086-1091. 10.1038/ncb1201-1086 11781570

[DMM050359C17] Homma, H., Tanaka, H., Jin, M., Jin, X., Huang, Y., Yoshioka, Y., Bertens, C. J. F., Tsumaki, K., Kondo, K., Shiwaku, H. et al. (2021). DNA damage in embryonic neural stem cell determines FTLDs’ fate via early-stage neuronal necrosis. *Life Sci. Alliance* 4, e202101022. 10.26508/lsa.202101022 34130995 PMC9715434

[DMM050359C18] Josephs, K. A. (2008). Frontotemporal dementia and related disorders: deciphering the enigma. *Ann. Neurol.* 64, 4-14. 10.1002/ana.21426 18668533

[DMM050359C19] Ju, J.-S., Miller, S. E., Hanson, P. I. and Weihl, C. C. (2008). Impaired protein aggregate handling and clearance underlie the pathogenesis of p97/VCP-associated disease. *J. Biol. Chem.* 283, 30289-30299. 10.1074/jbc.M805517200 18715868 PMC2573070

[DMM050359C20] Ju, J.-S., Fuentealba, R. A., Miller, S. E., Jackson, E., Piwnica-Worms, D., Baloh, R. H. and Weihl, C. C. (2009). Valosin-containing protein (VCP) is required for autophagy and is disrupted in VCP disease. *J. Cell Biol.* 187, 875-888. 10.1083/jcb.200908115 20008565 PMC2806317

[DMM050359C21] Kitamoto, T. (2001). Conditional modification of behavior in *Drosophila* by targeted expression of a temperature-sensitive *shibire* allele in defined neurons. *J. Neurobiol.* 47, 81-92. 10.1002/neu.1018 11291099

[DMM050359C22] Kondo, H., Rabouille, C., Newman, R., Levine, T. P., Pappin, D., Freemont, P. and Warren, G. (1997). p47 is a cofactor for p97-mediated membrane fusion. *Nature* 388, 75-78. 10.1038/40411 9214505

[DMM050359C23] Kushimura, Y., Tokuda, T., Azuma, Y., Yamamoto, I., Mizuta, I., Mizuno, T., Nakagawa, M., Ueyama, M., Nagai, Y., Yoshida, H. et al. (2018). Overexpression of *ter94*, *Drosophila VCP*, improves motor neuron degeneration induced by knockdown of *TBPH*, *Drosophila TDP-43*. *Am. J. Neurodegener. Dis.* 7, 11-31. 29531866 PMC5840287

[DMM050359C24] Li, N., Zhai, Y., Zhang, Y., Li, W., Yang, M., Lei, J., Tye, B.-K. and Gao, N. (2015). Structure of the eukaryotic MCM complex at 3.8 A. *Nature* 524, 186-191. 10.1038/nature14685 26222030

[DMM050359C25] Lin, M.-J., Cheng, C.-W. and Shen, C.-K. J. (2011). Neuronal function and dysfunction of Drosophila dTDP. *PLoS ONE* 6, e20371. 10.1371/journal.pone.0020371 21673800 PMC3105987

[DMM050359C26] Lukacsovich, T., Asztalos, Z., Juni, N., Awano, W. and Yamamoto, D. (1999). The *Drosophila melanogaster* 60A chromosomal division is extremely dense with functional genes: their sequences, genomic organization, and expression. *Genomics* 57, 43-56. 10.1006/geno.1999.5746 10191082

[DMM050359C27] Luo, L., Liao, Y. J., Jan, L. Y. and Jan, Y. N. (1994). Distinct morphogenetic functions of similar small GTPases: Drosophila Drac1 is involved in axonal outgrowth and myoblast fusion. *Genes Dev.* 8, 1787-1802. 10.1101/gad.8.15.1787 7958857

[DMM050359C28] Manno, A., Noguchi, M., Fukushi, J., Motohashi, Y. and Kakizuka, A. (2010). Enhanced ATPase activities as a primary defect of mutant valosin-containing proteins that cause inclusion body myopathy associated with Paget disease of bone and frontotemporal dementia. *Genes Cells* 15, 911-922. 10.1111/j.1365-2443.2010.01428.x 20604808

[DMM050359C29] Mimnaugh, E. G., Xu, W., Vos, M., Yuan, X. and Neckers, L. (2006). Endoplasmic reticulum vacuolization and valosin-containing protein relocalization result from simultaneous hsp90 inhibition by geldanamycin and proteasome inhibition by velcade. *Mol. Cancer Res.* 4, 667-681. 10.1158/1541-7786.MCR-06-0019 16966435

[DMM050359C30] Olney, N. T., Spina, S. and Miller, B. L. (2017). Frontotemporal dementia. *Neurol. Clin.* 35, 339-374. 10.1016/j.ncl.2017.01.008 28410663 PMC5472209

[DMM050359C31] Pfeffer, G., Lee, G., Pontifex, C. S., Fanganiello, R. D., Peck, A., Weihl, C. C. and Kimonis, V. (2022). Multisystem proteinopathy due to VCP mutations: a review of clinical heterogeneity and genetic diagnosis. *Genes (Basel)* 13, 963. 10.3390/genes13060963 35741724 PMC9222868

[DMM050359C32] Pintér, M., Jékely, G., Szepesi, R. J., Farkas, A., Theopold, U., Meyer, H. E., Lindholm, D., Nässel, D. R., Hultmark, D. and Friedrich, P. (1998). TER94, a *Drosophila* homolog of the membrane fusion protein CDC48/p97, is accumulated in nonproliferating cells: in the reproductive organs and in the brain of the imago. *Insect Biochem. Mol. Biol.* 28, 91-98. 10.1016/S0965-1748(97)00095-7 9639875

[DMM050359C33] Remus, D., Beuron, F., Tolun, G., Griffith, J. D., Morris, E. P. and Diffley, J. F. X. (2009). Concerted loading of Mcm2-7 double hexamers around DNA during DNA replication origin licensing. *Cell* 139, 719-730. 10.1016/j.cell.2009.10.015 19896182 PMC2804858

[DMM050359C34] Ritson, G. P., Custer, S. K., Freibaum, B. D., Guinto, J. B., Geffel, D., Moore, J., Tang, W., Winton, M. J., Neumann, M., Trojanowski, J. Q. et al. (2010). TDP-43 mediates degeneration in a novel *Drosophila* model of disease caused by mutations in VCP/p97. *J. Neurosci.* 30, 7729-7739. 10.1523/JNEUROSCI.5894-09.2010 20519548 PMC2890254

[DMM050359C35] Roy, L., Bergeron, J. J. M., Lavoie, C., Hendriks, R., Gushue, J., Fazel, A., Pelletier, A., Morré, D. J., Subramaniam, V. N., Hong, W. et al. (2000). Role of p97 and syntaxin 5 in the assembly of transitional endoplasmic reticulum. *Mol. Biol. Cell* 11, 2529-2542. 10.1091/mbc.11.8.2529 10930451 PMC14937

[DMM050359C36] Saito, M., Nakayama, M., Fujita, K., Uchida, A., Yano, H., Goto, S., Okazawa, H. and Sone, M. (2021). Role of the *Drosophila* YATA protein in the proper subcellular localization of COPI revealed by *in vivo* analysis. *Genes Genet. Syst.* 95, 303-314. 10.1266/ggs.20-00027 33583916

[DMM050359C37] Scarian, E., Fiamingo, G., Diamanti, L., Palmieri, I., Gagliardi, S. and Pansarasa, O. (2022). The role of VCP mutations in the spectrum of amyotrophic lateral sclerosis-frontotemporal dementia. *Front. Neurol.* 13, 841394. 10.3389/fneur.2022.841394 35273561 PMC8902152

[DMM050359C38] Shiraishi, R., Tamura, T., Sone, M. and Okazawa, H. (2014). Systematic analysis of fly models with multiple drivers reveals different effects of ataxin-1 and huntingtin in neuron subtype-specific expression. *PLoS ONE* 9, e116567. 10.1371/journal.pone.0116567 25551764 PMC4281079

[DMM050359C39] Sone, M., Uchida, A., Komatsu, A., Suzuki, E., Ibuki, I., Asada, M., Shiwaku, H., Tamura, T., Hoshino, M., Okazawa, H. et al. (2009). Loss of *yata*, a novel gene regulating the subcellular localization of APPL, induces deterioration of neural tissues and lifespan shortening. *PLoS ONE* 4, e4466. 10.1371/journal.pone.0004466 19209226 PMC2635962

[DMM050359C40] Sweeney, S. T., Hidalgo, A., de Belle, J. S. and Keshishian, H. (2012). Hydroxyurea ablation of mushroom bodies in Drosophila. *Cold Spring Harb. Protoc.* 2012, 231-234. 10.1101/pdb.prot067777 22301647

[DMM050359C41] Tang, W. K. and Xia, D. (2013). Altered intersubunit communication is the molecular basis for functional defects of pathogenic p97 mutants. *J. Biol. Chem.* 288, 36624-36635. 10.1074/jbc.M113.488924 24196964 PMC3868774

[DMM050359C42] Tang, W. K., Li, D., Li, C.-C., Esser, L., Dai, R., Guo, L. and Xia, D. (2010). A novel ATP-dependent conformation in p97 N-D1 fragment revealed by crystal structures of disease-related mutants. *EMBO J.* 29, 2217-2229. 10.1038/emboj.2010.104 20512113 PMC2905243

[DMM050359C43] Tresse, E., Salomons, F. A., Vesa, J., Bott, L. C., Kimonis, V., Yao, T.-P., Dantuma, N. P. and Taylor, J. P. (2010). VCP/p97 is essential for maturation of ubiquitin-containing autophagosomes and this function is impaired by mutations that cause IBMPFD. *Autophagy* 6, 217-227. 10.4161/auto.6.2.11014 20104022 PMC2929010

[DMM050359C44] Uchiyama, K., Jokitalo, E., Kano, F., Murata, M., Zhang, X., Canas, B., Newman, R., Rabouille, C., Pappin, D., Freemont, P. et al. (2002). VCIP135, a novel essential factor for p97/p47-mediated membrane fusion, is required for Golgi and ER assembly in vivo. *J. Cell Biol.* 159, 855-866. 10.1083/jcb.200208112 12473691 PMC2173386

[DMM050359C45] Wani, A., Zhu, J., Ulrich, J. D., Eteleeb, A., Sauerbeck, A. D., Reitz, S. J., Arhzaouy, K., Ikenaga, C., Yuede, C. M., Pittman, S. K. et al. (2021). Neuronal VCP loss of function recapitulates FTLD-TDP pathology. *Cell Rep.* 36, 109399. 10.1016/j.celrep.2021.109399 34289347 PMC8383344

[DMM050359C46] Watts, G. D. J., Wymer, J., Kovach, M. J., Mehta, S. G., Mumm, S., Darvish, D., Pestronk, A., Whyte, M. P. and Kimonis, V. E. (2004). Inclusion body myopathy associated with Paget disease of bone and frontotemporal dementia is caused by mutant valosin-containing protein. *Nat. Genet.* 36, 377-381. 10.1038/ng1332 15034582

[DMM050359C47] Weihl, C. C., Dalal, S., Pestronk, A. and Hanson, P. I. (2006). Inclusion body myopathy-associated mutations in p97/VCP impair endoplasmic reticulum-associated degradation. *Hum. Mol. Genet.* 15, 189-199. 10.1093/hmg/ddi426 16321991

[DMM050359C48] Weihl, C. C., Miller, S. E., Hanson, P. I. and Pestronk, A. (2007). Transgenic expression of inclusion body myopathy associated mutant p97/VCP causes weakness and ubiquitinated protein inclusions in mice. *Hum. Mol. Genet.* 16, 919-928. 10.1093/hmg/ddm037 17329348

[DMM050359C49] Wojcik, C., Yano, M. and DeMartino, G. N. (2004). RNA interference of valosin-containing protein (VCP/p97) reveals multiple cellular roles linked to ubiquitin/proteasome-dependent proteolysis. *J. Cell Sci.* 117, 281-292. 10.1242/jcs.00841 14657277

[DMM050359C50] Woodman, P. G. (2003). p97, a protein coping with multiple identities. *J. Cell Sci.* 116, 4283-4290. 10.1242/jcs.00817 14514884

[DMM050359C51] Xia, D., Tang, W. K. and Ye, Y. (2016). Structure and function of the AAA+ ATPase p97/Cdc48p. *Gene* 583, 64-77. 10.1016/j.gene.2016.02.042 26945625 PMC4821690

[DMM050359C52] Ye, Y., Meyer, H. H. and Rapoport, T. A. (2001). The AAA ATPase Cdc48/p97 and its partners transport proteins from the ER into the cytosol. *Nature* 414, 652-656. 10.1038/414652a 11740563

[DMM050359C53] Zhang, X., Gui, L., Zhang, X., Bulfer, S. L., Sanghez, V., Wong, D. E., Lee, Y., Lehmann, L., Lee, J. S., Shih, P.-Y. et al. (2015). Altered cofactor regulation with disease-associated p97/VCP mutations. *Proc. Natl. Acad. Sci. USA* 112, E1705-E1714. 10.1073/pnas.1418820112 25775548 PMC4394316

