# Supplementary material for: Loss of function of VCP/TER94 causes neurodegeneration
Source: Dis Model Mech. 2024 Dec 23;17(12):dmm050359. doi: 10.1242/dmm.050359 (PMC11698056; doi:10.1242/dmm.050359)
Supplement: Supplementary information [file dmm-17-050359-s1.pdf]

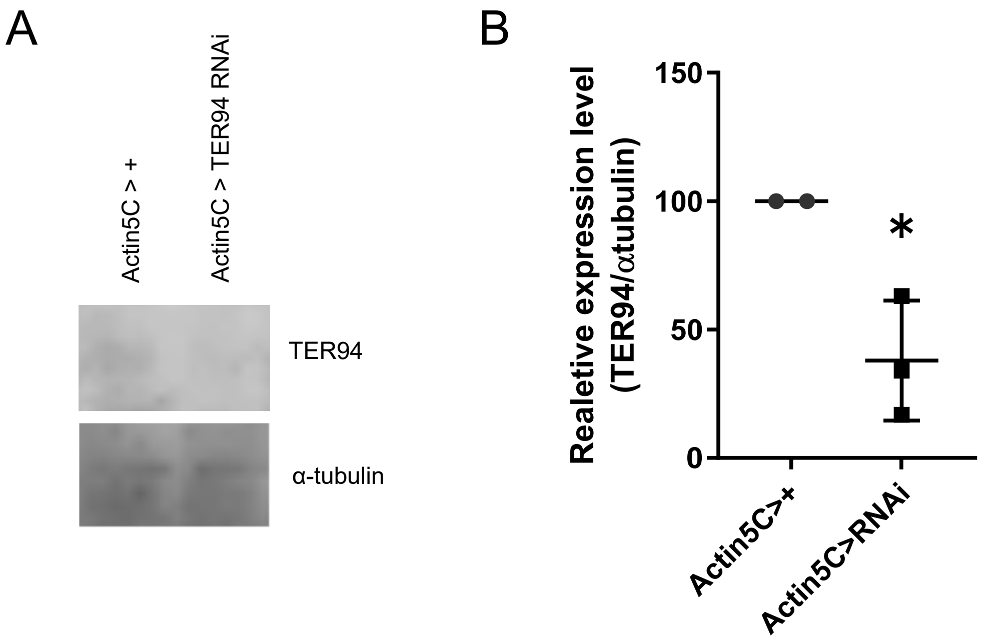

Fig. S1. Quantification of TER94 KD by western blot analysis. (A) Western blot analysis of lysates of whole first instar larvae using an antibody against TER94. (B) Quantification of the western blot data. The relative expression of TER94 normalized to the expression of α-tubulin is shown. The numbers of the examined data are 2 (control) and 3 (*TER94* RNAi). *: $p<0.05$ (t test).

Disease Models & Mechanisms • Supplementary information

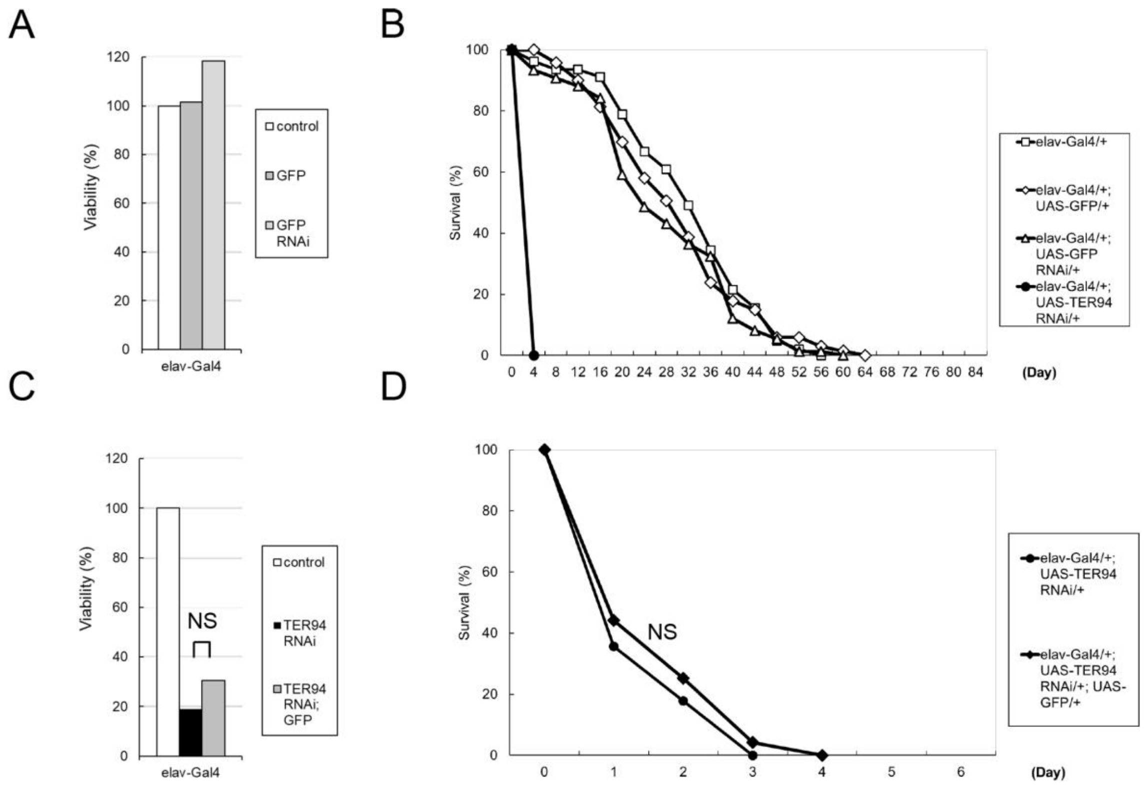

Fig. S2. Control experiments examining viability and lifespan. (A) Viability of flies during development from embryo to adult. Neither the neuronal expression of GFP nor its knockdown affected viability. The numbers of the examined flies are 293 (control), 108 (GFP) and 274 (GFP RNAi). (B) Lifespan after eclosion. Neither neuronal expression of GFP nor its knockdown did not affect lifespan. The numbers of the examined flies are 157 (control), 70 (GFP) and 76 (GFP RNAi). (C) Viability of flies during development from embryo to adult. Neuronal expression of GFP in the *TER94* KD flies did not affect viability. The numbers of the examined flies are 145 (*TER94* RNAi) and 147 (*TER94* RNAi; GFP). NS: not significant (chi-square test). (D) Lifespan after eclosion. Neuronal expression of GFP in *TER94* KD flies did not affect lifespan. The numbers of the examined flies are 14 (*TER94* RNAi) and 34 (*TER94* RNAi; GFP). NS: not significant (log rank test).

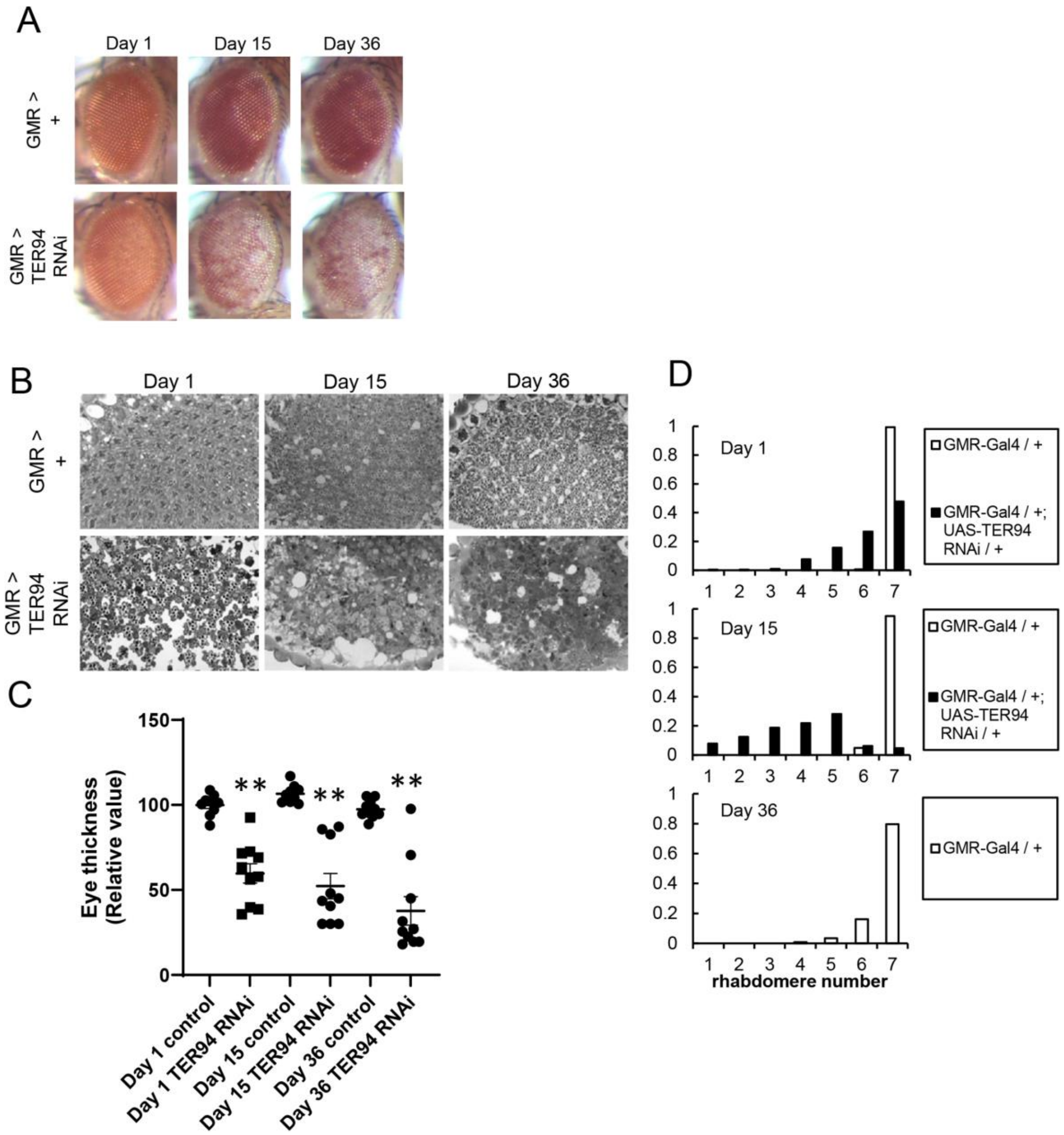

Fig. S3. Phenotypes caused by *TER94* KD in the compound eye. (A) External morphology of the compound eye. *TER94* KD caused progressive eye degeneration. The control images shown in Fig. S3A and Fig. S6 are the same, as data were collected simultaneously.(B) Internal morphology of the compound eye. 500 nm thick tangential semithin sections were stained with toluidine blue. *TER94* KD caused vacuolization and progressive deterioration of tissues. (C) Thickness of the compound eye determined from horizontal paraffin sections. *TER94* KD caused

Disease Models & Mechanisms • Supplementary information

a reduction in the thickness of the compound eye possibly as a result of degeneration. The numbers of the examined flies are 9 (Day 1, control), 10 (Day 1, *TER94* RNAi), 9 (Day 15, control), 10 (Day 15, *TER94* RNAi), 10 (Day 36, control), and 10 (Day 36, *TER94* RNAi). **: $p<0.01$ (t-test). (D) Progressive reduction in the number of rhabdomeres in an ommatidium. The numbers of the examined ommatidia are 183 (Day 1, control), 197 (Day 1, *TER94* RNAi), 144 (Day15, control), 64 (Day 15, *TER94* RNAi), 118 (Day 36, control), and 0 (Day 36, *TER94* RNAi).

Disease Models & Mechanisms • Supplementary information

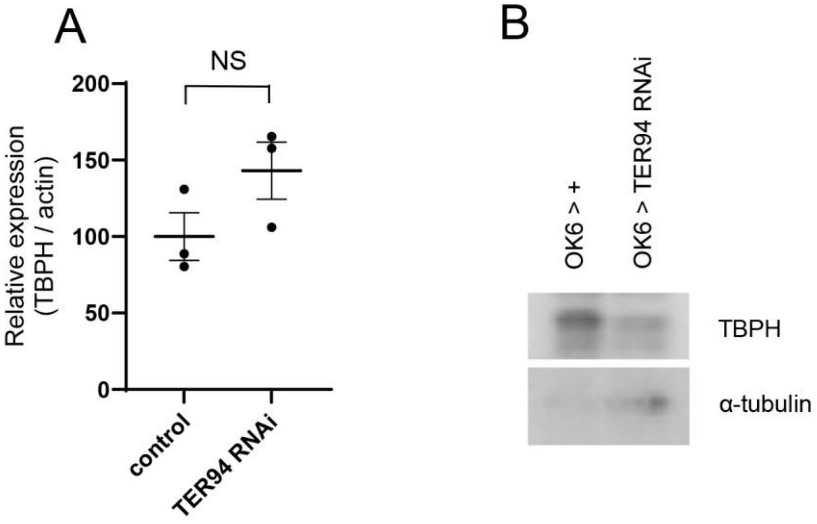

Fig. S4. Disappearance of TBPH in larval motor neurons with *TER94* KD. (A) The mRNA expression of *TBPH* normalized to that of *actin5C* mRNA was examined in larval brains by real-time PCR. *TBPH* mRNA expression was not significantly changed by *TER94* KD. The numbers of the examined samples are 3. (B) Western blot analysis of lysates of the anterior third of wandering third instar larvae containing the brain and the ventral nerve cord using an antibody against TBPH. The level of TBPH protein was decreased by *TER94* KD.

Disease Models & Mechanisms • Supplementary information

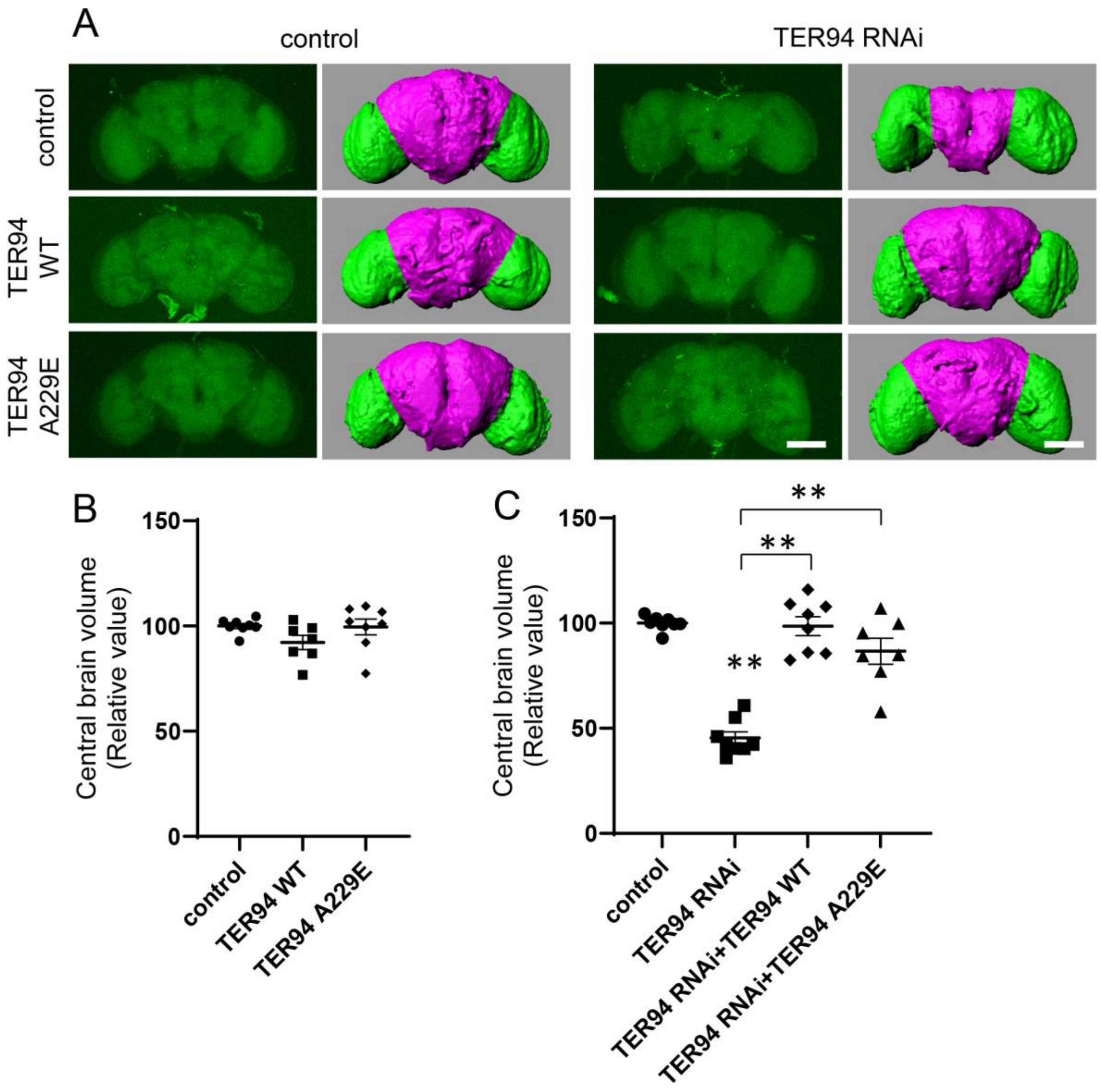

Fig. S5. Rescue experiment of reduced volume of the central brain of TER94 KD flies by wild-type and mutant TER94. (A) Morphology of the brains. The left panels show autofluorescence in projections of confocal images. The right panels are images reconstructed in Imaris software; the optic lobes are green and the central brain is magenta. The control images shown in Fig. 2A and Fig. S5A are the same, as data were collected simultaneously. Scale bars: 50 µm. (B) Central brain volume was not affected by neuronal expression of wild-type and mutant TER94. The numbers of the examined flies are 8 (control), 7 (TER94 WT), and 8 (TER94

Disease Models & Mechanisms • Supplementary information

A229E). (C) Neuronal expression of both wild-type and mutant *TER94* significantly rescued the reduction in central brain volume caused by *TER94* KD. The numbers of the examined flies are 8 (*TER94* RNAi), 8 (*TER94* RNAi; *TER94* WT), and 7 (*TER94* RNAi; *TER94* A229E). **: $p<0.01$ (Games-Howell test).

Disease Models & Mechanisms • Supplementary information

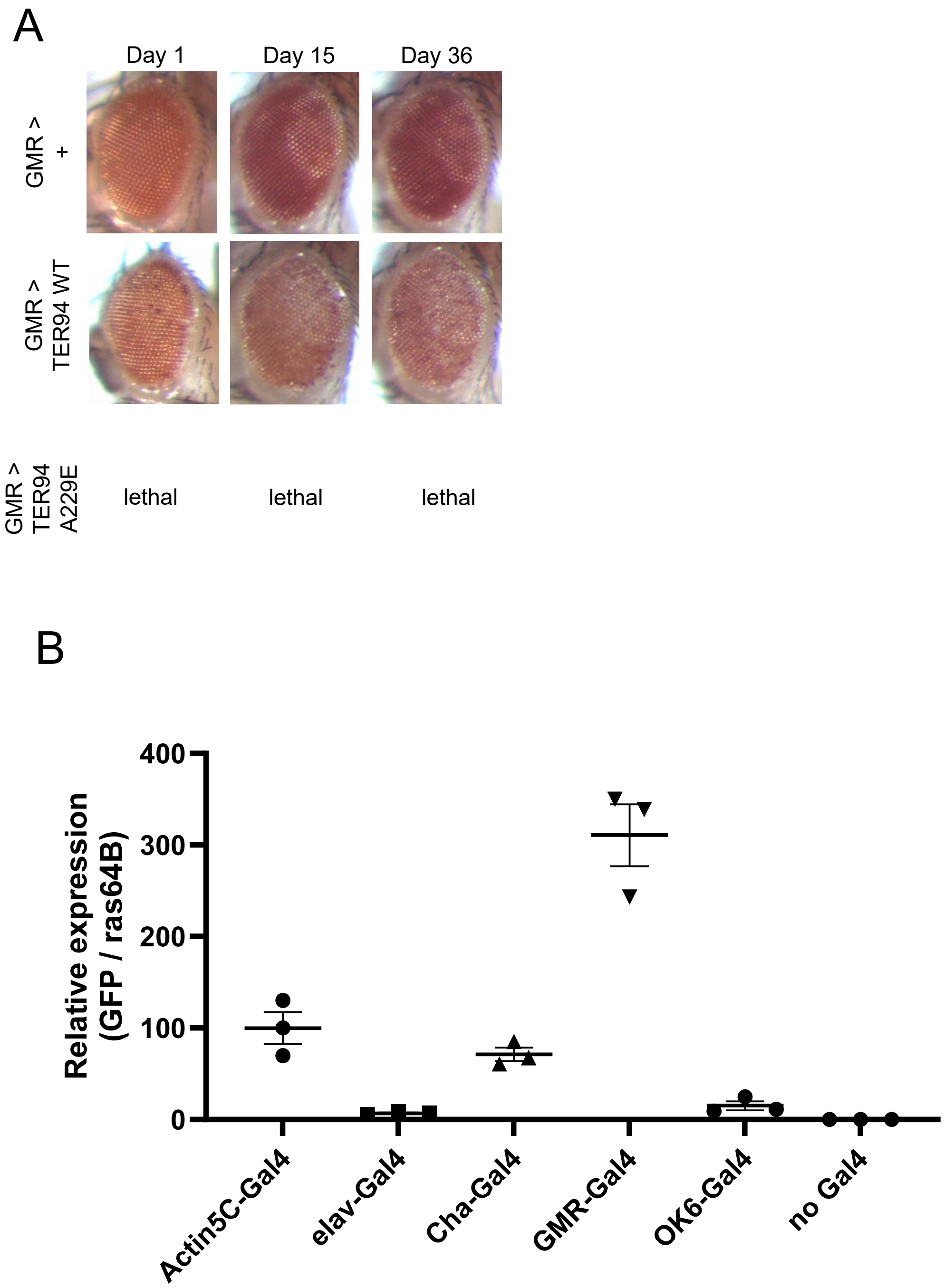

Fig. S6. Phenotypes caused by overexpression of TER94 in the compound eye. (A) External morphology of the compound eye. Overexpression of wild-type TER94 caused eye degeneration. Overexpression of A229E mutant TER94 caused lethality. The control images shown in Fig. S3A and Fig. S6 are the same, as data were collected simultaneously. (B) Quantification of expression induced by various Gal4 drivers**.** As a molecule that is not expressed endogenously, expression of GFP was induced, and its expression, which was normalized by expression of Ras64B, was quantified. Expression induced by elav-Gal4 was weak, whereas expression induced by other Gal4 drivers including GMR-Gal4 was far stronger. The numbers of the examined samples are 3.

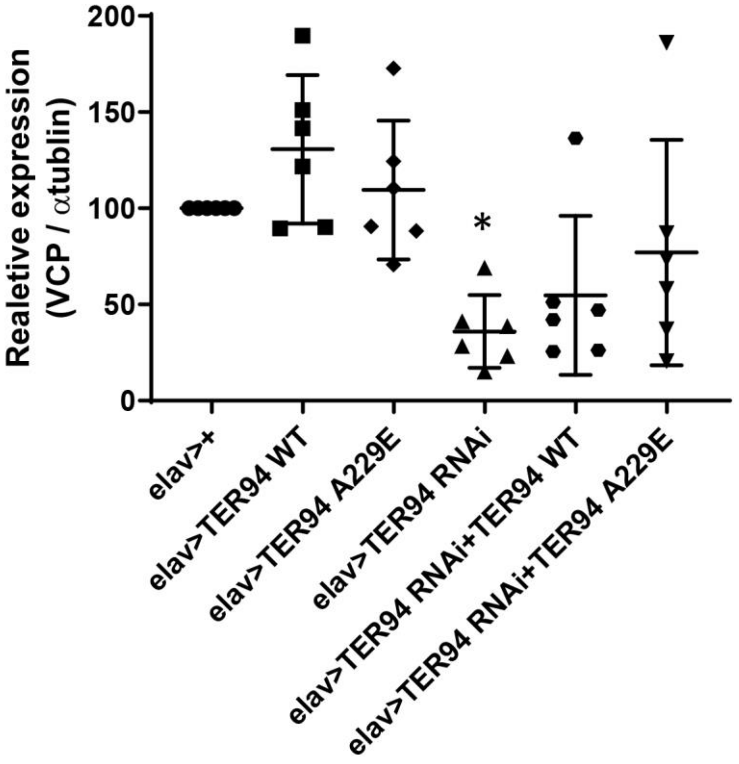

Fig. S7. Quantification of TER94 proteins in the genetic rescue experiment driven by elav-Gal4. The relative expression of TER94 proteins normalized to the expression of α-tubulin is shown. The number of the examined data is 6. *: $p<0.05$ (Dunnett test).

Disease Models & Mechanisms • Supplementary information

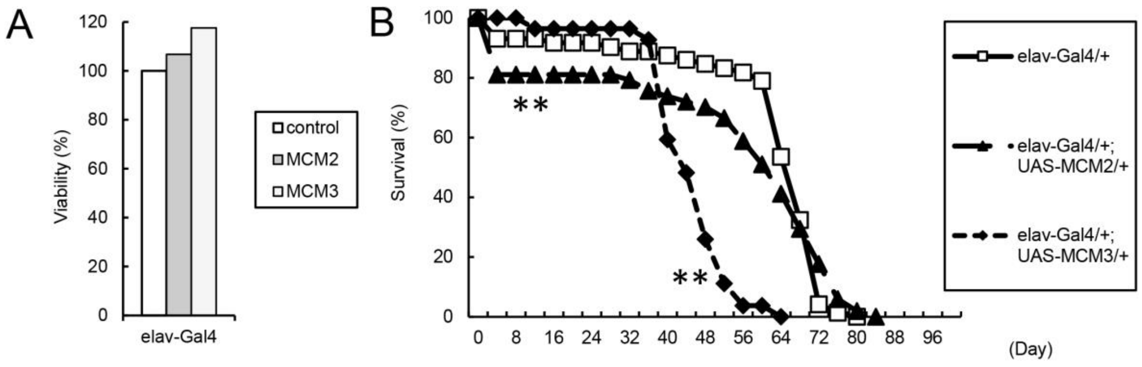

Fig. S8. Effect of overexpression of MCM2 or MCM3. (A) Viability of flies during development from embryo to adult. Neuronal expression of neither MCM2 nor MCM3 affected viability. The numbers of the examined flies are 347 (control), 279 (MCM2), and 106 (MCM3). (B) Lifespan after eclosion. Neuronal expression of MCM2 or MCM3 affected lifespan. The numbers of the examined flies are 72 (control), 58 (MCM2), and 28 (MCM3).

Disease Models & Mechanisms • Supplementary information

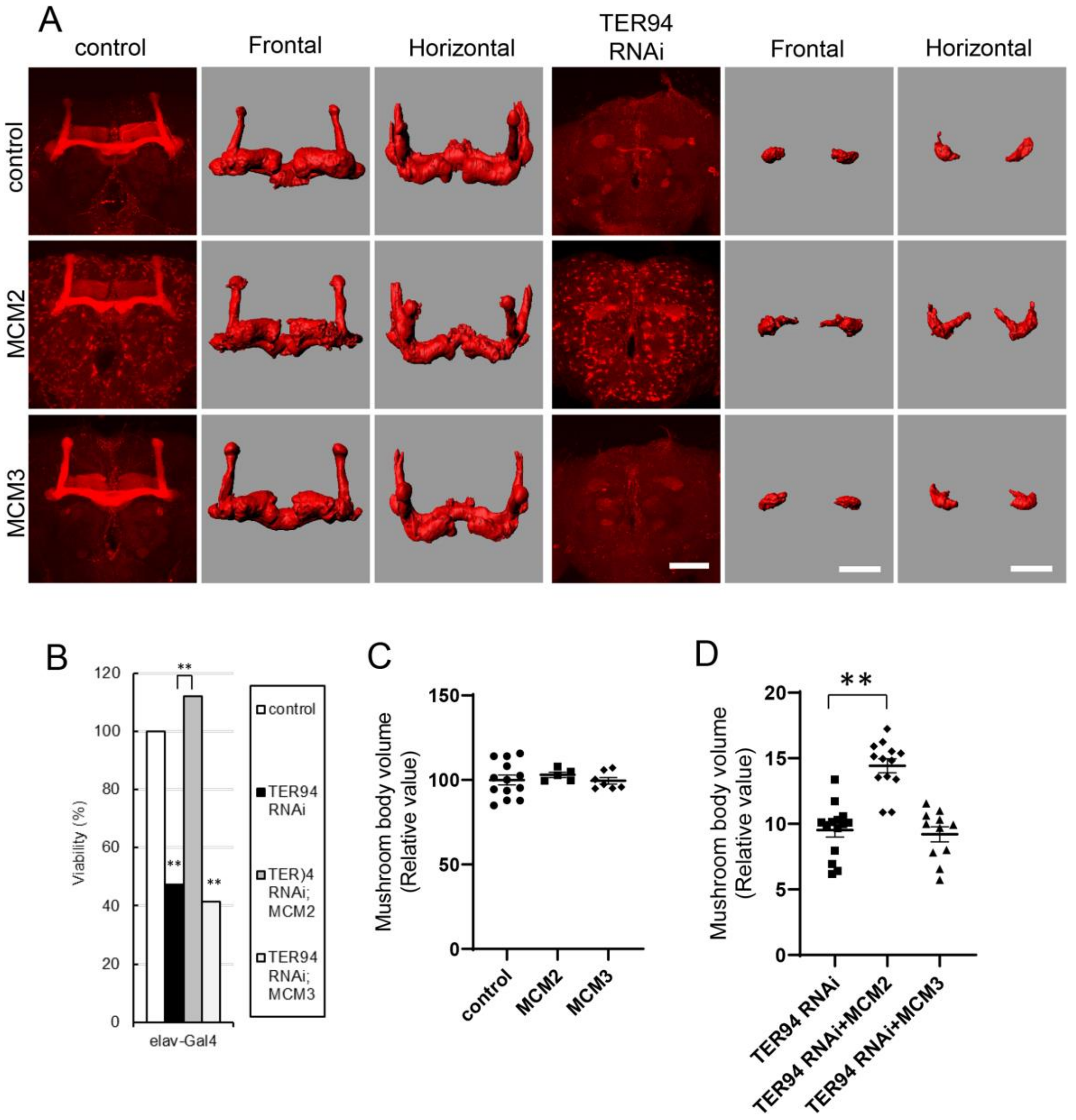

Fig. S9. Rescue effect of overexpression of MCM2 or MCM3. (A) Morphology of mushroom bodies on Day 1 after eclosion. The left panels are projections of confocal images stained by an anti-Fasciclin II antibody. The center and right panels are images reconstructed in Imaris software. The center panels are frontal images, and the right panels are horizontal images. The control images shown in Fig. 2B, Fig. 4A and Fig. S9A are the same, as data were collected simultaneously. Scale bar: 50 μm. (B) Viability of flies during development from embryo to adult. Expression of both

MCM2 rescued the reduction in viability caused by *TER94* KD, while expression of MCM3 did not rescue this change. The numbers of the examined flies are 784 (*TER94* RNAi), 1030 (*TER94* RNAi; MCM2), and 558 (*TER94* RNAi; MCM3). **: $p<0.01$ (chi-square test). (C) Mushroom body volume was not affected by neuronal expression of MCM2 and MCM3. The numbers of the examined flies are 13 (control), 5 (MCM2), and 7 (MCM3). (D) Neuronal expression of MCM2 significantly rescued the reduction in mushroom body volume caused by *TER94* KD, but expression of MCM3 did not rescue this change. The numbers of the examined flies are 14 (*TER94* RNAi), 13 (*TER94* RNAi; MCM2), and 11 (*TER94* RNAi; MCM3). **: $p<0.01$ (Dunnett test).

Disease Models & Mechanisms • Supplementary information

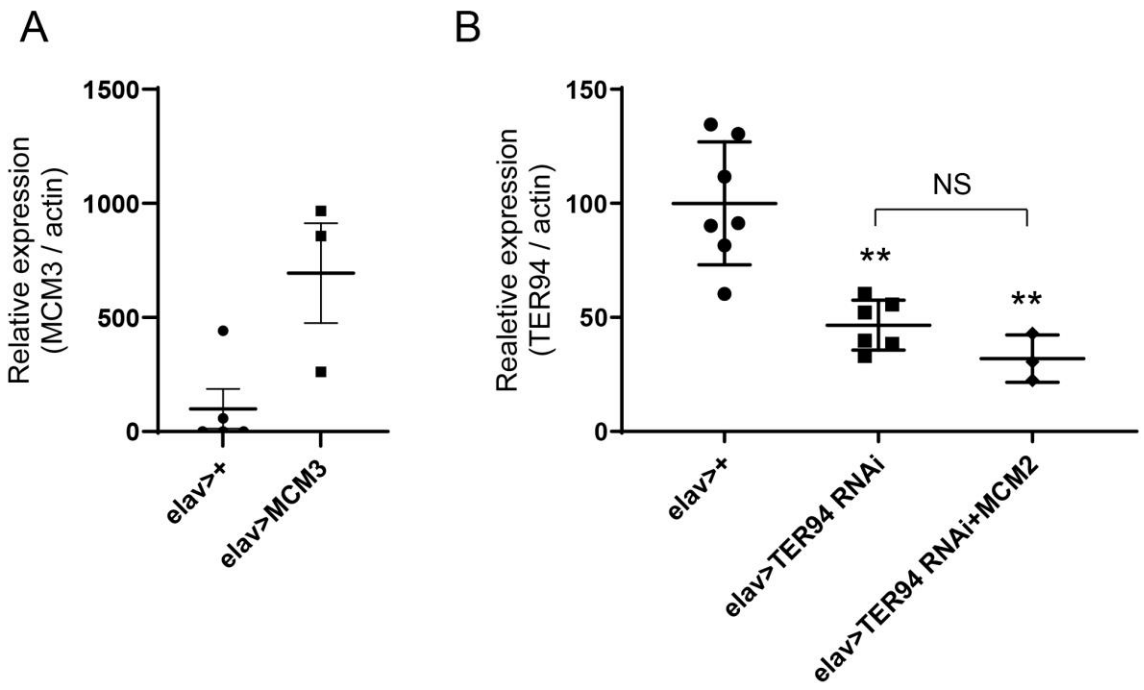

Fig. S10. Expression of *MCM3* and *TER94* in the genetic rescue experiments. (A) The mRNA expression of *MCM3* driven by elav-Gal4 was examined by real-time PCR and normalized to that of *actin5C* mRNA. The numbers of the examined samples are 5 (control) and 3 (MCM3). (B) The expression of *TER94* mRNA normalized to that of *actin5C* mRNA was examined. The decrease in *TER94* mRNA expression caused by *TER94* KD did not appear to be affected by the overexpression of MCM2. The numbers of examined samples are 7 (control), 6 (*TER94* RNAi) and 3 (*TER94* RNAi; MCM2). **: $p<0.01$ (Games-Howell test). NS: not significant.

Disease Models & Mechanisms • Supplementary information
